# Vegetation, pH and Water Content as Main Factors for Shaping Fungal Richness, Community Composition and Functional Guilds Distribution in Soils of Western Greenland

**DOI:** 10.3389/fmicb.2019.02348

**Published:** 2019-10-11

**Authors:** Fabiana Canini, Laura Zucconi, Claudia Pacelli, Laura Selbmann, Silvano Onofri, József Geml

**Affiliations:** ^1^Department of Ecological and Biological Sciences, University of Tuscia, Viterbo, Italy; ^2^Biodiversity Dynamics, Naturalis Biodiversity Center, Leiden, Netherlands; ^3^Section of Mycology, Italian National Antarctic Museum (MNA), Genoa, Italy; ^4^Faculty of Science, Leiden University, Leiden, Netherlands

**Keywords:** metabarcoding, functional guilds, shrub encroachment, edaphic factors, ITS1

## Abstract

Fungi are the most abundant and one of the most diverse components of arctic soil ecosystems, where they are fundamental drivers of plant nutrient acquisition and recycling. Nevertheless, few studies have focused on the factors driving the diversity and functionality of fungal communities associated with these ecosystems, especially in the scope of global warming that is particularly affecting Greenland and is leading to shrub expansion, with expected profound changes of soil microbial communities. We used soil DNA metabarcoding to compare taxonomic and functional composition of fungal communities in three habitats [bare ground (BG), biological soil crusts (BSC), and vascular vegetation (VV) coverage] in Western Greenland. Fungal richness increased with the increasing complexity of the coverage, but BGs and BSCs samples showed the highest number of unique OTUs. Differences in both fungal community composition and distribution of functional guilds identified were correlated with edaphic factors (mainly pH and water content), in turn connected with the different type of coverage. These results suggest also possible losses of diversity connected to the expansion of VV and possible interactions among the members of different functional guilds, likely due to the nutrient limitation, with potential effects on elements recycling.

## Introduction

Almost all plants are highly dependent on mutualistic relationships with mycorrhizal fungi, including ectomycorrhizae, ericoid and arbuscular mycorrhizae (Väre et al., 1992; Michaelson et al., 2008; Newsham et al., 2009) that can allow more efficient nutrient and water uptake (Gardes and Dahlberg, 1996; Hobbie and Hobbie, 2006; Hobbie et al., 2009; Bjorbækmo et al., 2010). Plants release many root compounds that are fundamental in selecting microorganisms colonizing the rhizosphere (Mendes et al., 2013); in the main time, endophytic and mycorrhizal fungi may promote plant growth (Mucciarelli et al., 2003) and their resistance to abiotic and biotic stress factors (Rodriguez et al., 2008). Moreover, degradation of organic matter predominantly relies on fungi that are the major drivers of nutrient recycling (Dahlberg and Bültmann, 2013). This is particularly true for cold and nutrient-poor arctic terrestrial environments, where fungi dominate the microbial biomass and play key roles in ecosystem functioning as symbionts (mycorrhizae, endophytes, and lichens), pathogens and decomposers (Gardes and Dahlberg, 1996; Hobbie et al., 2009; Newsham et al., 2009).

Given the above-mentioned intimate relationships of fungi and plants, many specific associations between both fungal and plant communities composition have been reported. The most relevant published studies indicated the importance of abiotic factors, such as climate at regional scale (Timling et al., 2012), edaphic factors and microclimate driven by micro- and mesotopography at landscape scale (Blaalid et al., 2014; Timling et al., 2014; Mundra et al., 2015; Geml et al., 2016; Grau et al., 2017). Among these mentioned works, Mundra et al. (2015) observed that fungal communities associated with *Bistorta vivipara* were driven by different factors, such as periglacial processes, more than the above-ground vegetation, while in the other studies the total soil fungal community composition seems to correlate with vegetation (or habitat) types.

Arctic regions have been experiencing some of the highest rates of warming (Turner et al., 2007), particularly evident in Greenland (Bevis et al., 2019), with an average increase of temperature of about 0.1°C per year since the 1980s (McBean et al., 2005). Temperature increase is influencing sea ice cover and the length of ice-free periods, resulting in an overall greening of terrestrial Arctic regions (Goetz et al., 2005; Bhatt et al., 2010). In particular, long-term experimental warming studies carried out in vegetation plots have indicated significant increases in the cover and height of shrubs (e.g., *Betula nana* and *Salix pulchra*), combined with a significant decrease of bryophytes and lichens (Mercado-Diaz, 2011; Pattison and Welker, 2014), most likely due to the competitive exclusion by shrubs (Cornelissen et al., 2001; Jägerbrand et al., 2009). Additionally, an analysis of the responses of tundra vegetation to experimental warming conditions across the Arctic suggested that these phenomena might continue in the future. In fact, abundance and height of shrubs have increased markedly in the last two decades, particularly in the Low Arctic (Elmendorf et al., 2012). These alterations are expected to be coupled with changes in soil fungal communities (Dahlberg and Bültmann, 2013), especially regarding mycorrhizal and root-associated fungi (Hobbie and Hobbie, 2006; Buckeridge and Grogan, 2008; Hobbie et al., 2009). Recently, several papers documented marked changes in the fungal community composition in long-term summer warming and increased snow pack (winter warming) experiments in the Arctic (Deslippe et al., 2011; Geml et al., 2015, 2016; Morgado et al., 2015, 2016; Semenova et al., 2015, 2016). While some compositional changes in soil fungal communities were likely caused by alterations in abiotic factors and edaphic processes, some trends, e.g., the decrease of lichen richness and abundance in the warmed plots, seemed to be a direct result of the increased growth of shrubs. In this regard, soil fungi could be viewed both as sentinels and as amplifiers of global change (Vincent, 2010). One of the most widespread hypotheses is that shifts in fungal communities composition are likely to affect carbon and nitrogen cycles in soil and promote the breakdown of organic matter (Clemmensen et al., 2006; Deslippe and Simard, 2011; Zhang et al., 2014; Treseder et al., 2016), increasing the release of greenhouse gases from soils to the atmosphere (Crowther et al., 2016; Commane et al., 2017), and consequently amplifying climate feedbacks (Zhou et al., 2009; Wieder et al., 2013; Abbott et al., 2016).

Knowledge on vegetation composition is fundamental to understand the functionality of any ecosystem, mainly for interspecific variations in productivity (Ward et al., 2013; Walker et al., 2015), root and litter inputs (Cornelissen et al., 2007; Ward et al., 2015), and plant–microbe associations (Read et al., 2004). For example, it has been hypothesized that the presence of different shrub species, such as *B. nana* (McLaren et al., 2017), characterized by rapid growth and easily decomposable litter, could lead to a faster carbon turnover rate (Ward et al., 2009, 2015).

In this paper, we compared richness and composition of functional guilds of soil fungal communities in three different habitats in West Greenland. The three habitats can be viewed as a gradient of vegetation complexity: bare ground (BG) without any apparent vegetation growth, biological soil crusts (BSCs) dominated by bryophytes and lichens, and soil covered with a vascular vegetation (VV), e.g., *Empetrum nigrum*, *Vaccinium uliginosum*, *B. nana*, and *Salix glauca*. In this context, the main aims were (i) to understand how differences in functional profiles of fungal communities relate to different habitats and to biotic and abiotic variables, and (ii) to gain insights into the landscape-level dynamics of vegetation and soil fungi in Western Greenland, in light of shrubs expansion, particularly *B. nana* (Hollesen et al., 2015).

## Materials and Methods

### Sampling Area

The study area was located in Kobbefjord, Nuuk, West Greenland (64°08′ N, 51°23′ W). The climate of the area was classified as low Arctic, subzone D (Jonasson et al., 2000). The mean annual air temperature of the area in the period 2008–2010 was 0.7°C and the mean air temperature of the warmest month, July, was 10.7°C. In winter, the average air temperatures was −30°C. The total annual precipitation was 838–1127 mm and an average of 25–50% of the total annual precipitation fell as snow during the winter period (Søndergaard et al., 2012).

Sampling was carried out in July 27–31, 2017 in an area close the NERO line (Bay et al., 2008), a vegetation transect where plant compositions have been monitored for more than 10 years. In total, twenty 2-m^2^ plots scattered in the landscape, representing the three above-mentioned habitat types, were sampled: 5 in BGs, 6 in BSCs, and 9 in VVs. BG and BSC plots were generally adjacent to the vegetation and in some cases small patches were dispersed among the vegetation. Exact coordinates, elevation, and shrub genera composition for each plot are listed in Supplementary Table S1. In each plot, three replicates of soil samples were collected aseptically at a depth of 5 cm, after removing the top of the soil, resulting in a total of 60 samples. Samples were stored at −20°C in sterile bags until molecular analyses.

### Soil Characteristics

Soil water content was measured with a gravimetric method, starting from 5 g of soil (Reynolds, 1970) and measuring the weight before and after drying in oven, repeating the measurements on dried samples until no variation in weight was observed. pH was measured in a 1:2.5 suspension of dried soil in deionized water, with a HI9321 pH meter (Hanna Instruments Woonsocket, Rhode Island, United States). For each sample, water content and pH were measured in independent triplicates and the mean of the three measurements was considered for the final value.

Phosphorous (P), Carbon (C) and Nitrogen (N) content analyses have been carried out at Eszterházy Károly University, Eger, Hungary. P content was measured via Microwave Plasma Atomic Emission Spectrometry (MP-AES) and C and N content via CNS elemental analyzer.

### DNA Extraction, Amplification, and Sequencing

For each sample, metagenomic DNA was extracted from 0.5 g of soil using DNEasy Powersoil kit (QIAGEN, Hilden, Germany), according to the manufacturer’s protocol. The ITS1 region was PCR amplified using ITS1F (Gardes and Bruns, 1993) and ITS2 (White et al., 1990) primers as described in Smith and Peay (2014). The equimolar pool of uniquely barcoded amplicons was paired-end sequenced (2 × 300 bp) on an Illumina MiSeq platform at the Vincent J. Coates Genomics Sequencing Laboratory at University of California, Berkeley.

### Bioinformatic Analyses

Bcl files were converted to Fastq files and were demultiplexed and primer were removed using bcl2fastq (v 2.18). Dual-matched 8-bp indexes were used to eliminate the occurrence of “barcode bleed” (or tag-switching) between samples.

ITS1 demultiplexed sequences were processed with the Amplicon ToolKit (AMPtk) for NGS data (formally UFITS) v.1.2.1 (Palmer et al., 2018). 6 813 346 starting reads were subjected to quality trimming and PhiX screening using USEARCH v. 9.2.64 (Edgar, 2010) with default parameters. Reads with less than 100 bp were removed, reads longer than 300 bp were trimmed and paired-end reads were merged in one step. We obtained 3 405 965 quality-filtered contigs. Individual sample sequence files were merged into a single file and clustered into Operational Taxonomic Units (OTUs) with a 97% identity threshold using VSEARCH v. 2.7.0 (Rognes et al., 2016), simultaneously removing putative chimeras. A total of 3 129 891 (92%) reads have been mapped in 3 491 OTUs. Singletons and rare OTUs (<5 reads) were removed as recommended by Lindahl et al. (2013), resulting in 2 938 OTUs retained. We assigned OTUs to taxonomic groups based on the curated UNITE + INSD reference database dynamic Species Hypotheses (SH) (UTAX release of October 10, 2017) using USEARCH v. 9.2.64 (Edgar, 2010). OTUs with <70% identity to a fungal sequence were excluded from the following analyses, resulting in 2661 OTUs retained. Representative sequences of fungal OTUs have been submitted to GenBank (BioProject PRJNA526618). The OTU table was normalized for subsequent statistical analyses by rarefying the number of high-quality fungal sequences to the smallest library size (33 870 reads) using the rrarefy function implemented in the vegan package v. 2.5-2 (Oksanen et al., 2018) in R (R Core Team, 2018). Only OTUs with >90% similarity to a fungal SH with known ecological functions were assigned to one of the following functional guilds: animal pathogens, ectomycorrhizal (ECM) fungi, ericoid mycorrhizal (ERM) fungi, lichenized fungi, mycoparasites, plant pathogens, other root-associated fungi (non-ECM fungi, non-ERM fungi, and root endophytes) and saprotrophs. The initial functional assignments were made by FunGuild (Nguyen et al., 2016) and manually checked afterward based on ecological metadata of the corresponding SHs in UNITE for genera that are known to comprise species with diverse functions.

### Statistical Analyses

Unless otherwise specified, all analyses were carried out with the vegan package v. 2.5-2 (Oksanen et al., 2018) in R (R Core Team, 2018). Total fungal richness, richness of functional guilds and their relative abundances were compared among the three habitats using ANOVA and Tukey’s HSD test. In addition, linear regression analyses were used to examine relationships between the above mentioned edaphic factors and richness of the total community, of the functional guilds, and their relative abundance. Linear regression was also used to examine the relations among the community composition differences of the total fungal community and of the components of the eight functional guilds (Bray-Curtis distance between pair of samples of Hellinger-transformed OTU table) and the corresponding differences in the soil parameters. We ran non-metric multidimensional scaling (NMDS) on the Hellinger-transformed OTU table. Ordinations were run separately for functional guilds as well as for all fungi with the following specifications: distance measure = Bray-Curtis, dimensions = 2, initial configurations = 100, model = global, maximum number of iterations = 200, convergence ratio for stress = 0.999999. We used the *envfit* R function to fit edaphic variables and the relative abundance of the shrub genera (*Betula*, *Empetrum*, *Salix*, and *Vaccinium*; Supplementary Table S1) onto the NMDS ordinations. In addition, we tested whether fungal communities were statistically different among habitat types using the multi response permutation procedure (MRPP) and we determined any preference of individual OTUs for each habitat using indicator species analysis (Dufrêne and Legendre, 1997) in PC-ORD v. 6.0 (McCune et al., 2002).

Permutational multivariate analysis of variance (PerMANOVA; Anderson, 2001) was carried out on Bray-Curtis distance matrices obtained of Hellinger-transformed OTU tables with 9999 permutations, with the *adonis* function, in order to determine the effect of each soil physico-chemical characteristic, as well as the type of coverage (BG, BSC or VV), on the observed variance of the total community and of the functional guilds identified. To account for correlations among environmental variables, we performed a forward selection of parameters, including only significant environmental variables in the final models. The same approach was used also taking into account only 27 samples of VV plots in order to assess the effect of the edaphic characteristics, and the relative abundance of the four dominant shrub genera (*Salix*, *Betula*, *Vaccinium*, and *Empetrum*), on the variance of the total community and of the different functional guilds.

## Results

### Fungal Richness and Abundance Patterns

The quality-filtered and rarefied dataset contained 2661 fungal OTUs. The proportions of OTUs found exclusively in VV was the highest (23.6%) compared to BSC and BG samples (7.8 and 16%, respectively), whereas the BG samples showed the highest number of indicator OTUs (266 OTUs, compared with 146 OTUs for VV and 215 for BSC samples, respectively; Supplementary Table S2).

**FIGURE 1:**
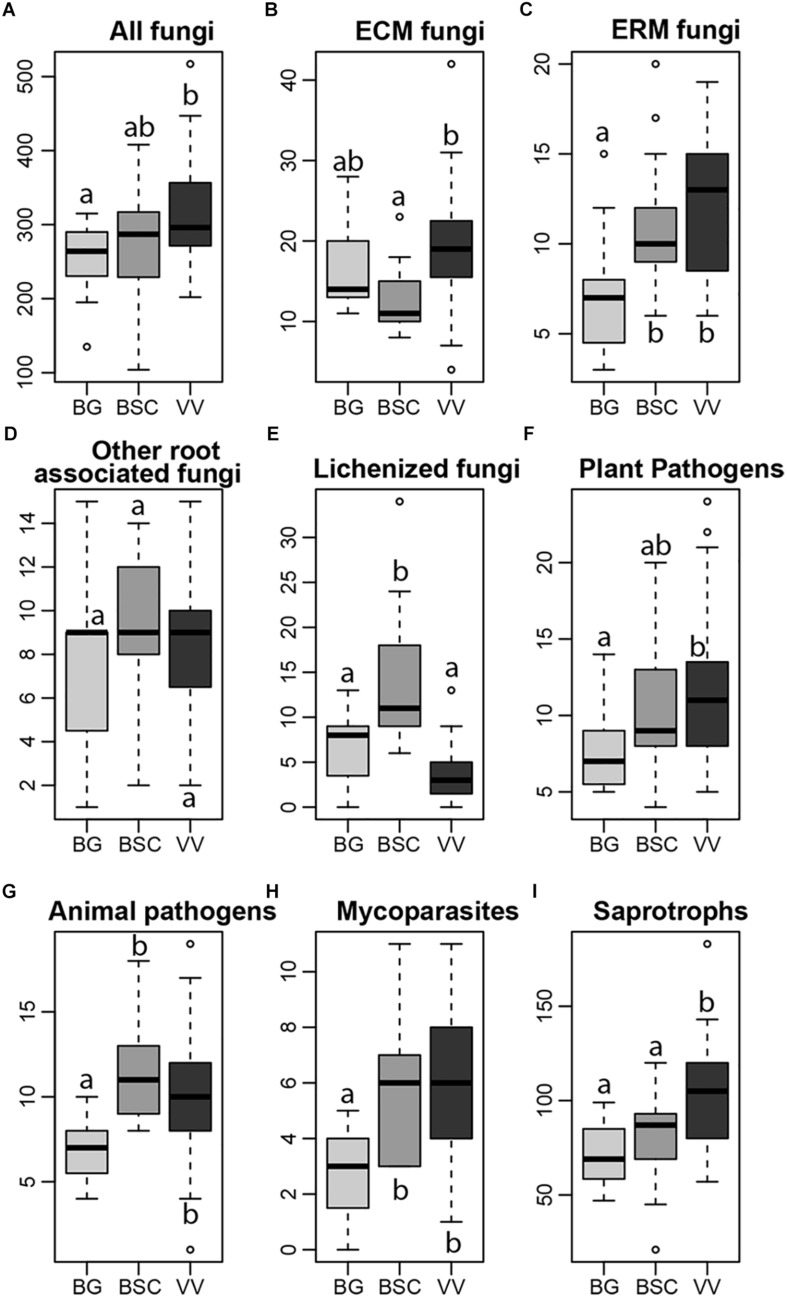
Richness of the total fungal communities **(A)** and of ectomycorrhizal (ECM), ericoid mycorrhizal (ERM), other root associated, lichenized, plant pathogenic, animal pathogenic, mycoparasites and saprotrophic fungi **(B–I)**, in each habitat (light gray, Bare Grounds (BG) plots; dark gray, Biological Soil Crusts (BGS) plots; black, Vascular Vegetation (VV) plots). Letters indicate significant differences in one-way ANOVA *post hoc* Tukey HSD test (significant for *p* < 0.05).

The richness of the total fungal community showed a slight increase ranging from BG to vegetation covered soil plots (Figure 1A). This trend seemed to be driven mainly by Ascomycota, the most abundant phylum (1303 OTUs, 49% of the total) in BSC samples, instead VV samples had a significative higher proportion of Basidiomycota, the second most abundant phylum (775 OTUs, 30% of the total) (Supplementary Figure S1).

With respect to functional assignments, 1105 OTUs (41.53% of the rarefied dataset) had >90% similarity to a fungal SH with known ecological function and were assigned to functional guilds. Five out of the eight functional guilds examined, namely ERM fungi, mycoparasites, plant pathogens, animal pathogens and saprotrophic fungi, showed an increase in richness from BG to VV plots (Figures 1C,F–I). Among these guilds, the increase of richness in VV plots respect to BSCs was significant only for saprotrophs. ECM fungi showed an increase of richness in VV plots in respect to BSCs, but a not significant difference between VV and BG plots (Figure 1B). For the remaining root associated fungi, we did not find significant differences in richness among the three habitats (Figure 1D). As expected, lichenized fungi showed the highest richness in BSC samples (Figure 1E), where bryophytes and lichens are dominant. When significant, the trends were the same also for the relative abundance of the components of the guilds (Supplementary Figure S2), except for root-associated fungi (not significant for the richness) that showed an increase from BG to VV and BSC plots (Supplementary Figure S2C).

### Correlation Between Richness and Relative Abundance of Fungal Guilds to Soil Parameters

**FIGURE 2:**
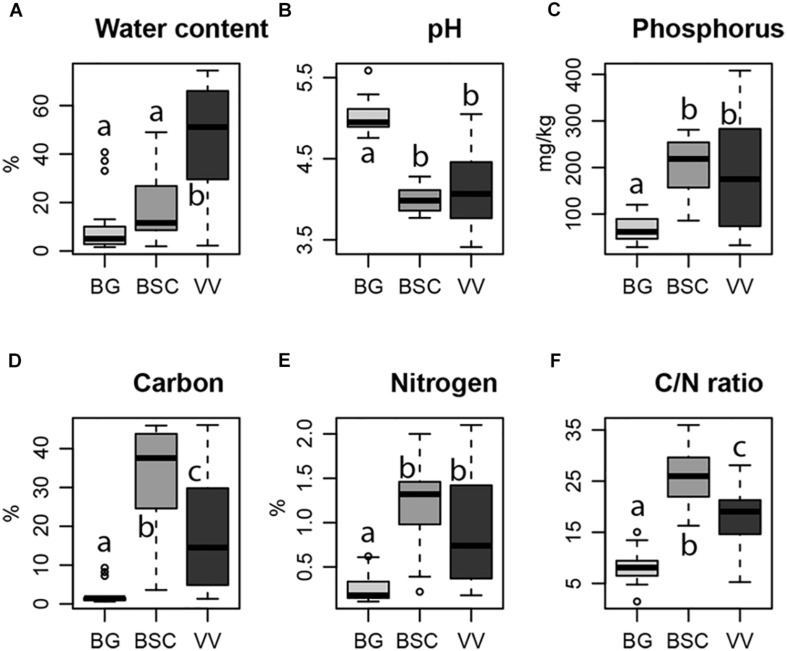
Variation of soil water content **(A)**, pH **(B)**, phosphorus content **(C)**, carbon content **(D)**, nitrogen content **(E)** and C/N ratio **(F)** in each habitat considered (light gray, Bare Ground (BG) plots; dark gray, Biological Soil Crusts (BSC) plots; black, Vascular Vegetation (VV) plots). Letters indicate significant differences in one-way ANOVA *post hoc* Tukey HSD test (significant for *p* < 0.05).

Edaphic parameters measured had significant differences among the three habitats, with the water relative content increasing from BG samples to BSC and VV plots and the pH showing an opposite trend, with the highest values in BG samples (Figures 2A,B). An increasing trend from BG to BSC and VV plots has been shown for the P, C, and N content, and for the ratio of these last two (Figures 2C–F). However, C content and C/N ratio were higher in BSC plots compared to VV ones.

**FIGURE 3:**
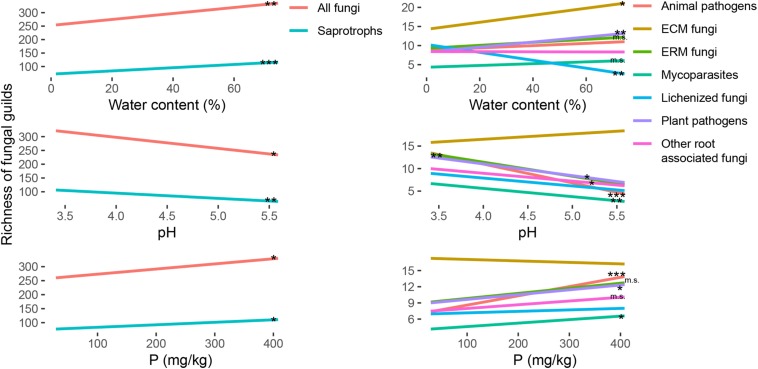
Regression lines for the variation of richness (*y*-axis) of the total fungal community and of ectomycorrhizal (ECM), ericoid mycorrhizal (ERM), other root associated, lichenized, plant pathogenic, animal pathogenic, mycoparasites and saprotrophic fungi, in response to soil parameters (water content, pH and P content; *x*-axis). The significance of the regressions is indicated as ^∗∗∗^*p* < 0.001, ^∗∗^*p* < 0.01, ^∗^*p* < 0.05, m. s. (marginally significant) *p* < 0.1. Single graphs of the regressions with the points corresponding to all the samples are reported in Supplementary Figure S3. All the slopes and *r*^2^ values for statistically significant regressions are reported in Supplementary Table S3.

**FIGURE 4:**
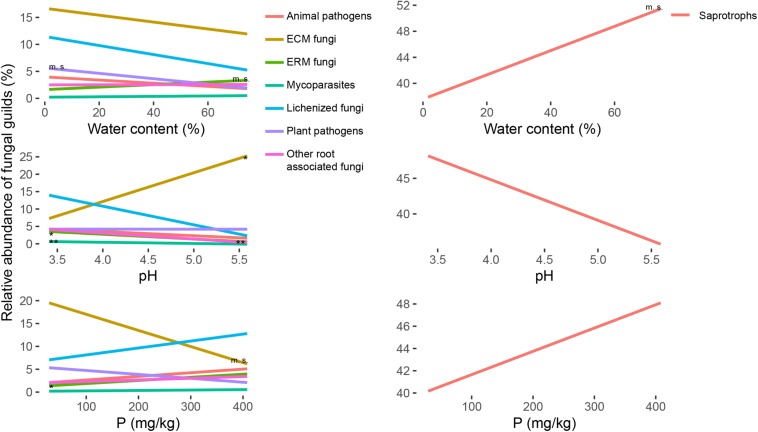
Regression lines for the variation of relative abundance (*y*-axis) of ectomycorrhizal (ECM), ericoid mycorrhizal (ERM), other root associated, lichenized, plant pathogenic, animal pathogenic, mycoparasites and saprotrophic fungi, in response to soil parameters (water content, pH and P content; *x*-axis). The significance of the regressions is indicated as ^∗∗^*p* < 0.01, ^∗^*p* < 0.05, m. s. (marginally significant) *p* < 0.1. Single graphs of the regressions with the points corresponding to all the samples are reported in Supplementary Figure S4. All the slopes and *r*^2^ values for statistically significant regressions are reported in Supplementary Table S3.

The richness of the fungal communities had a significative positive correlation with the water content of the soil samples (Figure 3 and Supplementary Table S3). The same trend was significant also for many fungal functional guilds, as ECM fungi, ERM fungi, plant pathogens, mycoparasites and saprotrophs, and was the opposite for lichenized fungi that showed a lower diversity for the wettest samples (Figure 3 and Supplementary Table S3). The same trends were marginally significant only for the abundance of ERM fungi and saprotrophs, and opposite in respect to the trend of plant pathogens abundance (Figure 4 and Supplementary Table S3).

Regarding the effect on the richness of the total fungal community, the increasing pH had a negative impact, with a decrease of biodiversity (Figure 3 and Supplementary Table S3). Going deeper in the different guilds (Figure 3), the richness for ERM fungi, root associated fungi, plant pathogens, animal pathogens, mycoparasites and saprotrophs was negatively correlated with the pH (Supplementary Table S3). Regarding the abundance (Figure 4), the same trend was also significant for ERM fungi, root associated fungi, and mycoparasites (Supplementary Table S3). ECM fungi abundance was positively correlated with soil pH (Supplementary Table S3), but their richness was not. Total richness was positively correlated with phosphorus content also (Figure 3 and Supplementary Table S3), as well as the richness of many guilds: ERM fungi, root associate fungi, animal pathogens, mycoparasites, and saprotrophs (Supplementary Table S3). Among the functional guilds, ERM fungi abundance was marginally negatively correlated with P content (Figure 4 and Supplementary Table S3).

The content of C and N and their ratio were generally positively correlated with the richness (Supplementary Figure S3), except for ECM, but the correlations were significative for few groups only. Animal pathogens showed the strongest effects of these parameters on their richness (Supplementary Figure S3 and Supplementary Table S3). ERM fungi richness was marginally correlated with these three parameters (Supplementary Figure S3 and Supplementary Table S3). N content had a marginally significant effect on mycoparasites and saprotrophs, and for this two guilds and lichenized fungi C/N ratio was significant (Supplementary Table S3). With respect to the abundance, root associated fungi (ECM, ERM and other root associated) were generally correlated with these three parameters, negatively for ECM and positively for the other two guilds (Supplementary Figure S4 and Supplementary Table S3). The abundance correlation was less strong for animal pathogens (Supplementary Figure S4 and Supplementary Table S3). In general, even been significant with 95% confidence, all the regressions had low *r*^2^ values and have to be carefully considered for precise predictions.

### Fungal Community Composition

**FIGURE 5:**
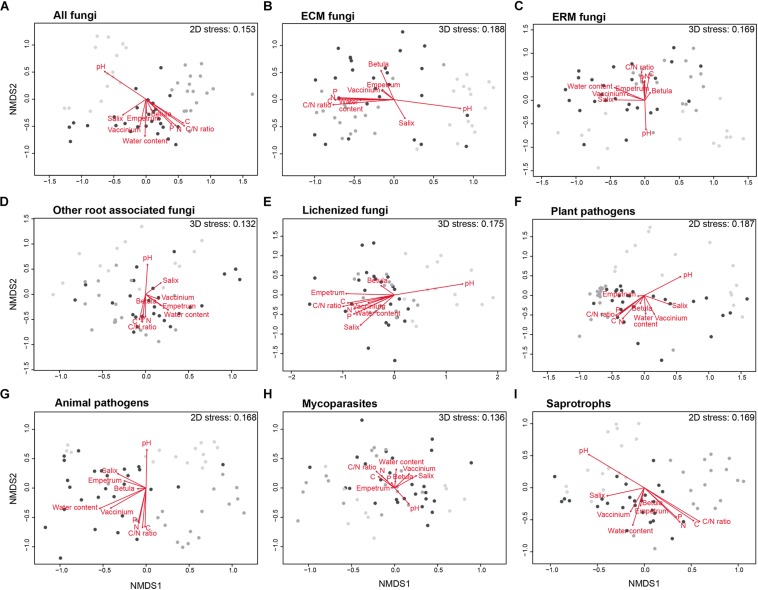
Non-metric multidimensional scaling (NMDS) ordinations of the differences (Bray–Curtis distance) in composition of fungal communities (Hellinger transformed OTUs abundances) in the habitats studied (light gray, Bare Ground (BG) plots; dark gray, Biological Soil Crusts (BSC) plots; black, Vascular Vegetation (VV) plots) for the total fungal community **(A)** and for ectomycorrhizal (ECM), ericoid mycorrhizal (ERM), other root associated, lichenized, mycoparasites, animal pathogens, plant pathogens and saprotrophic fungi **(B–I)**. The significance and the strength of the correlation of all the variables in the figure is reported in Supplementary Table S4.

The composition of the total fungal community changed significantly among the three different habitats, as visualized in the NMDS ordination (Figure 5A), resulted in a two-dimensional final solution with a stress value of 0.153. The MRPP analysis revealed that the difference was statistically supported (A = 0.082, *p* = 0.001).

For some of the functional guilds identified in the community, the NMDS analyses resulted in a final 2-dimensional ordination with stress values of 0.168 for animal pathogens, 0.187 for plant pathogens and 0.169 for saprotrophic fungi. For the other guilds, a 3-dimensional solution was necessary, resulting in final stress values of 0.188 for ECM fungi, 0.169 for ERM fungi, 0.132 for root associated fungi, 0.175 for lichenized fungi, and 0.136 for mycoparasites. In all cases, the NMDS ordinations (Figure 5) showed a strong structuring of fungal communities according to the different habitats, as confirmed by the MRPP analyses, that revealed a significative difference in the distribution among the three different habitats (*p* = 0.001), with the strongest effects for animal and plant pathogens (A = 0.154 and A = 0.107, respectively). All the other guilds showed less strong diversification among the habitats (A = 0.079 for ECM fungi, A = 0.086 for ERM fungi, A = 0.066 for root-associated fungi, A = 0.050 for lichenized fungi, A = 0.045 for mycoparasites, and A = 0.084 for saprotrophs). pH and C/N ratio resulted to be the strongest two parameters for the community composition for almost all the guilds (Supplementary Table S4).

### Effects of Edaphic Parameters and Type of Coverage on Fungal Community Composition

In order to assess the effect of the soil physico-chemical characteristics on fungal community structure, the differences in community composition (Bray-Curtis distance) were plotted against the differences in the single soil parameters. All regressions were highly significant (Supplementary Figure S5).

PerMANOVA analysis was used to infer the degree to which edaphic parameters and the presence/absence and type of vegetation coverage could explain the variance in community composition among samples. When considering the available parameters independently, all were significant in determining the structure of the fungal communities, even at the level of every single functional guild identified (except water content for lichenized fungi; Table 1). The type of habitat (BG, BSC, or VV) resulted to be the strongest parameter, followed by the pH in all the guilds, except animal pathogens (Table 2). When combined, depending on their influence, C and P content were not independent from the other parameters in shaping the total community. The type of habitat explained by itself more than 18% of the variance for the total community and was dominant for all the groups, with the highest percentages for plant and animal pathogens (20.03 and 28.07%, respectively; Table 2). The second dominant parameter acting independently for almost all the guilds (except lichenized fungi and mycoparasites) was the pH. Nitrogen content also was an independent driver of total community composition and of many guilds, including ECM fungi and saprotrophs. Finally, water content, even being a determinant parameter for the total community, was significant only for ERM fungi among functional guilds.

**TABLE 1:** Proportion of variation in fungal community composition, explained by soil variables calculated independently with permutational multivariate analysis of variance, based on Hellinger-transformed fungal community matrices.

**Variable**	**All fungi**		**ECM fungi**		**ERM fungi**		**Other root associated fungi**		**Lichenized fungi**	
	**Variance (%)**	***p***	**Variance (%)**	***p***	**Variance (%)**	***p***	**Variance (%)**	***p***	**Variance (%)**	***p***
Habitat	18.145	0.001	16.549	0.001	17.028	0.001	14.083	0.001	11.713	0.001
pH	10.267	0.001	10.259	0.001	8.836	0.001	9.974	0.001	6.063	0.001
Water content	6.325	0.001	5.634	0.001	5.913	0.001	4.845	0.005	2.708	0.093
C	7.705	0.001	8.796	0.001	6.127	0.001	6.683	0.001	4.460	0.003
N	6.508	0.001	7.638	0.001	5.227	0.002	5.456	0.001	3.878	0.006
C/N ratio	9.315	0.001	9.591	0.001	8.198	0.001	7.673	0.001	5.285	0.002
P	5.968	0.001	7.632	0.001	4.683	0.002	5.666	0.003	3.341	0.019

**Variable**	**Plant pathogens**		**Animal pathogens**		**Mycoparasites**		**Saprotrophs**			
	**Variance (%)**	***p***	**Variance (%)**	***p***	**Variance (%)**	***p***	**Variance (%)**	***p***		

Habitat	20.030	0.001	28.070	0.001	11.041	0.001	18.464	0.001		
pH	14.895	0.001	11.731	0.001	6.659	0.001	10.734	0.001		
Water content	4.116	0.020	12.803	0.001	4.763	0.006	6.376	0.001		
C	9.780	0.001	11.712	0.001	4.601	0.012	8.624	0.001		
N	7.910	0.001	10.162	0.001	4.332	0.017	7.126	0.001		
C/N ratio	11.346	0.001	13.548	0.001	5.219	0.002	10.315	0.001		
P	7.303	0.001	8.271	0.002	3.987	0.025	6.199	0.001		

*Significant variables were included in the final model for each fungal guild (Table 2).*

**TABLE 2:** Proportion of variation in fungal community composition, at level of the total community and the eight functional guilds, explained by soil variables added sequentially (from the first to the last) in a model, depending on their independent influence in the variance, as reported in Table 1.

**All fungi**			**ECM fungi**			**ERM fungi**			**Other root associated fungi**			**Lichenized fungi**		
**Variable**	**Variance (%)**	***p***	**Variable**	**Variance (%)**	***p***	**Variable**	**Variance (%)**	***p***	**Variable**	**Variance (%)**	***p***	**Variable**	**Variance (%)**	***p***
Habitat	**18.145**	**0**.**0001**	Habitat	**16.549**	**0**.**0001**	Habitat	**17.028**	**0**.**0001**	Habitat	**14.083**	**0**.**0001**	Habitat	**11.713**	**0**.**0001**
pH	**3.697**	**0**.**0004**	pH	**4.230**	**0**.**0002**	pH	**3.063**	**0**.**0055**	pH	**3.547**	**0**.**0179**	pH	1.781	0.3655
C/N ratio	**2.886**	**0**.**0017**	C/N ratio	2.015	0.0769	C/N ratio	**2.464**	**0**.**0319**	C/N ratio	0.927	0.7922	C/N ratio	2.005	0.2446
C	1.355	0.3958	C	1.460	0.3682	C	1.395	0.4009	C	0.614	0.9429	C	0.798	0.9625
N	**3.708**	**0**.**0001**	N	**2.495**	**0**.**0158**	Water content	**4.655**	**0**.**0001**	P	0.782	0.8698	N	**4.126**	**0**.**0010**
Water content	**2.119**	**0**.**0302**	P	**2.170**	**0**.**0448**	N	1.846	0.1547	N	2.748	0.0667	P	2.153	0.1775
P	1.779	0.1001	Water content	2.066	0.0680	P	1.927	0.1217	Water content	0.653	0.9278	Residuals	77.424	
Residuals	66.311		Residuals	69.015		Residuals	67.622		Residuals	76.647				

**Plant pathogens**			**Animal pathogens**			**Mycoparasites**			**Saprotrophs**					
**Variable**	**Variance (%)**	***p***	**Variable**	**Variance (%)**	***p***	**Variable**	**Variance (%)**	***p***	**Variable**	**Variance (%)**	***p***			

Habitat	**20.030**	**0**.**0001**	Habitat	**28.070**	**0**.**0001**	Habitat	**11.041**	**0**.**0001**	Habitat	**18.464**	**0**.**0001**			
pH	**4.366**	**0**.**0027**	C/N ratio	**5.213**	**0**.**0012**	pH	2.717	0.0822	pH	**3.914**	**0**.**0003**			
C/N ratio	2.027	0.1156	Water content	2.292	0.0508	C/N ratio	1.606	0.3841	C/N ratio	**2.926**	**0**.**0032**			
C	2.475	0.0574	pH	**4.840**	**0**.**0004**	Water content	2.864	0.0645	C	1.639	0.1706			
N	**3.681**	**0**.**0086**	C	1.173	0.3555	C	1.705	0.3514	N	**4.030**	**0**.**0003**			
P	2.225	0.0813	N	1.784	0.1266	N	0.474	0.9611	Water content	2.031	0.0527			
Water content	0.964	0.6422	P	1.766	0.1339	P	**4.528**	**0**.**0044**	P	1.811	0.0994			
Residuals	64.232		Residuals	54.862		Residuals	75.066		Residuals	65.186				

*Significant values in bold.*

### Effect of Shrub Composition on Fungal Communities

The effect of the relative abundance of shrubs genera in VV plots (Supplementary Table S1) was addressed via PerMANOVA analysis. In this case, all the parameters were significant in shaping the total community, but very few of them were determinant for many of the functional guilds (Table 3). In particular, for ECM fungi two shrub genera (*Vaccinium* and *Empetrum*) were not significant, and for other root associated fungi only *Salix* was determinant for the observed variance. Combining the variables, it resulted that *Salix* relative abundance was the main driver of VV plots communities (Table 4), explaining the highest percentages of variance for all the guilds except ECM fungi, that were surprisingly mainly driven by abiotic parameters (pH and water content). Instead, ERM fungi were mainly shaped by shrub genera abundance, even if *Salix* effect was dominant in respect to *Vaccinium* and *Empetrum* (Table 4). For saprotrophic fungi also, the presence of different types of shrub was the main determinant factor for community composition.

**TABLE 3:** Proportion of variation in fungal community composition of vascular vegetation (VV) plots explained by soil variables and shrub genera relative abundances, calculated independently with permutational multivariate analysis of variance, based on Hellinger-transformed fungal community matrices.

**Variables**	**All fungi**		**ECM fungi**		**ERM fungi**		**Other root associated fungi**		**Lichenized fungi**	
	**Variance (%)**	***p***	**Variance (%)**	***p***	**Variance (%)**	***p***	**Variance (%)**	***p***	**Variance (%)**	***p***
pH	**10.417**	**0**.**001**	**11.492**	**0**.**001**	**8.675**	**0**.**003**	**10.924**	**0**.**003**	4.526	0.418
Water content	**7.925**	**0**.**003**	**12.691**	**0**.**001**	**6.999**	**0**.**014**	2.451	0.756	2.310	0.920
C	**7.878**	**0**.**007**	**11.698**	**0**.**001**	**6.344**	**0**.**036**	5.265	0.193	2.750	0.833
N	**7.346**	**0**.**004**	**11.272**	**0**.**001**	**6.283**	**0**.**042**	3.983	0.401	2.274	0.917
C/N ratio	**6.838**	**0**.**008**	**10.272**	**0**.**001**	5.617	0.082	5.220	0.207	3.619	0.665
P	**7.070**	**0**.**008**	**11.002**	**0**.**001**	**6.335**	**0**.**040**	3.720	0.476	2.557	0.890
*Salix*	**13.155**	**0**.**001**	**10.657**	**0**.**001**	**10.549**	**0**.**001**	**21.695**	**0**.**001**	**9**.**625**	**0**.**003**
*Betula*	**6.723**	**0**.**010**	**7.588**	**0**.**008**	**7.827**	**0**.**008**	3.294	0.550	4.610	0.395
*Vaccinium*	**6.245**	**0**.**018**	5.581	0.099	**7.093**	**0**.**015**	1.715	0.865	3.949	0.573
*Empetrum*	**8.435**	**0**.**002**	5.837	0.060	**9.910**	**0**.**001**	6.650	0.092	4.951	0.324

**Variables**	**Plant pathogens**		**Animal pathogens**		**Mycoparasites**		**Saprotrophs**			
	**Variance (%)**	***p***	**Variance (%)**	***p***	**Variance (%)**	***p***	**Variance (%)**	***p***		

pH	**10.017**	**0**.**014**	**7.786**	**0**.**027**	**7.861**	**0**.**024**	**11.430**	**0**.**001**		
Water content	6.280	0.107	**9.035**	**0**.**012**	4.485	0.318	**7.627**	**0**.**008**		
C	**9.737**	**0**.**017**	**8.175**	**0**.**034**	5.754	0.127	**8.114**	**0**.**002**		
N	**8.341**	**0**.**036**	6.849	0.071	5.697	0.138	**7.575**	**0**.**007**		
C/N ratio	4.995	0.221	6.795	0.061	4.817	0.255	**7.089**	**0**.**012**		
P	6.044	0.096	6.144	0.079	5.173	0.183	**7.368**	**0**.**007**		
*Salix*	**16.513**	**0**.**001**	**10.254**	**0**.**010**	**9.268**	**0**.**022**	**13.505**	**0**.**001**		
*Betula*	4.401	0.291	4.808	0.231	3.835	0.419	**6.468**	**0**.**025**		
*Vaccinium*	**15.556**	**0**.**001**	**7.826**	**0**.**029**	6.010	0.132	**6.642**	**0**.**021**		
*Empetrum*	**12.262**	**0**.**002**	7.064	0.051	**7.670**	**0**.**026**	**9.157**	**0**.**002**		

*Significant variables (in bold) were included in the final model for each fungal guild (Table 4).*

**TABLE 4:** Proportion of variation in fungal community composition of vascular vegetation (VV) plots, at level of the total community and the eight functional guilds explained by shrub genera abundance and soil variables.

**All fungi**			**ECM fungi**			**ERM fungi**			**Other root associated fungi**		
**Variable**	**Variance (%)**	***p***	**Variable**	**Variance (%)**	***p***	**Variable**	**Variance (%)**	***p***	**Variable**	**Variance (%)**	***p***
*Salix*	**13.155**	**0**.**0001**	Water content	**12.691**	**0**.**0001**	*Salix*	**10.549**	**0**.**0001**	*Salix*	**21.695**	**0**.**0003**
pH	**6.262**	**0**.**0007**	C	**6.349**	**0**.**0031**	*Empetrum*	**9.203**	**0**.**0001**	pH	1.671	0.8237
*Empetrum*	**7.244**	**0**.**0003**	pH	**7.384**	**0**.**0001**	pH	**6.415**	**0**.**0011**	Residuals	76.634	
Water content	**7.981**	**0**.**002**	N	4.108	0.0917	*Betula*	**5.726**	**0**.**0050**			
C	**4.174**	**0**.**0354**	P	4.130	0.0864	*Vaccinium*	**6.189**	**0**.**0021**			
N	**4.266**	**0**.**0340**	*Salix*	**6.374**	**0**.**0021**	Water content	**7.324**	**0**.**0003**			
P	3.121	0.2533	C/N ratio	2.791	0.4924	C	2.955	0.3461			
C/N ratio	2.982	0.3097	*Betula*	**4.731**	**0**.**0342**	P	4.031	0.0808			
*Betula*	3.995	0.0548	Residuals	51.442		N	2.099	0.7252			
*Vaccinium*	3.023	0.2889				Residuals	45.510				
Residuals	43.796										

**Plant pathogens**			**Animal pathogens**			**Mycoparasites**			**Saprotrophs**		
**Variable**	**Variance (%)**	***p***	**Variable**	**Variance (%)**	***p***	**Variable**	**Variance (%)**	***p***	**Variable**	**Variance (%)**	***p***

*Salix*	**16.513**	**0**.**0001**	*Salix*	**10.254**	**0**.**0023**	*Salix*	**9.268**	**0**.**0135**	*Salix*	**13.505**	**0**.**0001**
*Vaccinium*	**13.659**	**0**.**0001**	Water content	**9.049**	**0**.**0020**	pH	4.086	0.3051	pH	**6.671**	**0**.**0005**
*Empetrum*	3.399	0.2546	C	**8.039**	**0**.**0037**	*Empetrum*	6.560	0.0514	*Empetrum*	**7.920**	**0**.**0002**
pH	4.385	0.1226	*Vaccinium*	**7.460**	**0**.**0066**	Residuals	80.086		C	**4.655**	**0**.**0196**
C	4.182	0.1527	pH	7.417	0.0082				Water content	**6.664**	**0**.**0006**
N	2.809	0.3981	Residuals	57.781					N	**5.193**	**0**.**0071**
Residuals	55.053								P	2.559	0.5066
									C/N ratio	3.664	0.1034
									*Vaccinium*	3.183	0.2203
									*Betula*	3.311	0.1822
									Residuals	42.676	

*Terms were added sequentially (first to the last) in a model, depending on their independent influence in the variance, as reported in Table 3. Significant values in bold.*

## Discussion

This study is the first to characterize fungal communities in a Western Greenland landscape. Data presented here reveal that fungal community composition, as well as richness and relative abundance of functional guilds clearly differ among the three sampled habitats. Such strong structuring at small spatial scales confirms patterns observed in Eastern Greenland (Grau et al., 2017), Svalbard (Blaalid et al., 2014; Mundra et al., 2015), and the North American Arctic (Timling et al., 2014; Geml et al., 2016). However, the observed positive relationship between fungal richness and vegetation complexity differs from previous findings in Eastern Greenland, where, in the corresponding heath tundra, the BG habitat had the highest fungal richness, significantly higher than habitats dominated by *Dryas* and *Salix* (Grau et al., 2017). In our study, BG plots, showing the lowest richness, had a high percentage (16%) of exclusive OTUs and the highest number of indicator species (Supplementary Table S2). In spite of the differences in richness patterns among habitats, the high number of taxa specific to bare soils is a remarkable common feature with the study of Grau et al. (2017) and confirms that the harshest habitats in the Arctic appear to harbor a unique set of stress-tolerant fungi that likely are outcompeted in more vegetated habitat types. As also stated by Grau et al. (2017), the expected expansion of shrub dominated communities to previously unvegetated areas could lead to the loss of fungal diversity, specific of habitats without vegetation cover.

Community composition appeared to be mainly shaped by the type of habitat and by differences in abiotic conditions connected to the habitats. In particular, among the edaphic parameters tested, pH differed among habitats and correlated strongly with fungal richness and abundance, and community composition. Similar trends for soil pH values have been reported in primary successional chronosequences at glacier fronts, where young soils (bare soils), containing a relatively low amount of organic matter and clay minerals, had higher pH than older soils covered by biological crusts and then by shrubs (Kwon et al., 2015). Soil pH has been reported as one of the strongest parameters in shaping the fungal communities across the Arctic (Timling et al., 2014). In particular, pH variations, resulting in different plant communities, have been observed to be the major drivers at the transition between the bioclimatic subzones D and E in northern Alaska, where the main differences in fungal communities were observed (Timling et al., 2014). A strong effect of pH on the community structure has been observed also for soil bacteria across different biomes in the same regions (Chu et al., 2010; Siciliano et al., 2014).

The observed increase in P content with increased vegetation complexity in our plots (Figure 2) is similar to what was already reported for a glacier foreland (Borchhardt et al., 2019), where microbial P storage increased with distance from the glacier. Despite being abundant in the topsoil of bedrock of glaciated regions due to mineral weathering (Bradley et al., 2014), P is subjected to high rates of release by leaching (Wu et al., 2015). Microbial assimilation and storage of P is fundamental for its availability for plant colonization. The possible increasing in plant coverage could increase fungal richness and this could explain the strong positive relationship of P content and fungal richness that we found. Even being significant for community composition, in most cases P effect was not independent from other parameters. P availability is highly affected by soil pH, the main determinant of our communities. In fact, P is highly soluble at neutral pH values and becomes less available at basic and acidic pH levels. Similar effects for soil P content have been reported also for root associated fungi in the high Arctic, making it difficult to disentangle the effects of pH from those of other edaphic factors (Fujimura and Egger, 2012).

With respect to functional guilds, richness and relative abundance of ECM fungi were higher in the VV plots, likely due to increased abundance of their hosts. However, both richness and relative abundance of ECM fungi were surprisingly high also in BG plots, with no significant difference compared to VV plots, being lower only in BSC samples (Figure 1 and Supplementary Figure S2). Root associated fungi have shown to be highly diverse already in recently exposed areas in the Arctic (Blaalid et al., 2012) and ECM fungi in the Arctic bioclimatic subzone A, lacking of woody species and characterized by fine-grained soils, occasionally covered by lichens, bryophytes, cyanobacteria, and scattered forbs (Timling et al., 2014). In first instance, this situation could be explained by the fact that in the earliest stages of fungal colonization and communities development, stochastic processes are the main driving factors of communities composition (Jumpponen, 2003), favored by the already well documented high dispersal ability of Arctic fungi (Geml et al., 2012), of which ECM fungi are the main components. Some ECM sequences found in these plots could originate from spores, which could facilitate the expansion of shrubs from the surrounding areas. However, it is remarkable that the composition of ECM fungal communities in the BGs was well differentiated from the communities in the vegetated plots, suggesting a negligible effect of ‘spore rain’. Therefore, a probable explanation for the high prevalence of ECM fungi in the BG plots is that most of them are likely associated with the roots of shrubs and other hosts growing in adjacent vegetated patches, present in BG samples as small fragments. Furthermore, the compositional differences among habitats likely indicate the influence of edaphic factors on the community composition of ECM fungi, associated with the root system of the same host, at small spatial scales. For example, parts of a shrub root system may grow in soils with different pH, which is expected to shape ECM community composition. Additionally, taking into account only VV plots, we found that pH and water content had a stronger effect on ECM community composition than above shrub composition, confirming the importance of differences in edaphic conditions in shaping ECM fungal community structure at small spatial scales. Finally, the negative relationship between soil N content and ECM fungi richness and abundance has been repeatedly reported (Bödeker et al., 2014), which likely explains at least in part the patterns observed here, i.e., the lower abundance and richness of ECM fungi in BSC plots, characterized by higher N content.

ERM fungi showed an increase in richness with increased vegetation coverage, likely due to the presence of their host species (Figure 1). Their diversity and, to a lesser extent, their abundance were also high in BSC plots. ERM fungi have evolved recently from saprotrophic fungi (Martino et al., 2018) and, unlike ECM fungi that, during the transition to fully mycorrhizal habit, have lost genes coding for plant cell wall-degrading enzymes (Martin et al., 2016), all ERM fungi, regardless of their taxonomic position, feature a large set of degrading enzymes, specifically those involved in the degradation of hemicelluloses, pectins, glucans and mannans. This suggests that they still are in a transitional evolutionary stage between saprotrophy and mutualism (Martino et al., 2018) and, thus, are not obligate mutualists. Therefore, ERM fungi can occur in BSC plots, where their symbiotic hosts are not present and where they can survive as saprotrophs, thanks to their ability to thrive in more limiting conditions. Among the habitats sampled in our study, ERM fungal community composition was mainly driven by edaphic variables. They are usually characteristic of acidic soils, with low nutrients availability and high content of recalcitrant compounds (Cairney and Meharg, 2003); the strong negative correlations between the richness and relative abundance of ERM fungi and soil pH confirm that. Additionally, in the VV plots, we found that despite the presence of two Ericaceae genera being significant in shaping the structure of ERM communities, the effect of the *Salix* relative abundance was stronger. This pattern may be explained by the versatile habit of ERM fungi as well, because, besides being able to act as decomposers, ERM fungi can also live as endophytes in root tips of ECM plants (Bergero et al., 2000; Tedersoo et al., 2009; Grelet et al., 2010; Kernaghan and Patriquin, 2011; Vohník et al., 2013), and colonize both co-occurring ECM and non-ECM species (Chambers et al., 2008).

Saprotrophic fungi had a higher richness and relative abundance in VV plots, accounting also for the great part of indicator OTUs with an assigned function (33.6% of the OTUs identified as indicator species for this habitat). Their greater richness and relative abundance in the vegetated plots is likely due, in part, to greater soil moisture (Supplementary Figure S3) and possibly to an increase in plant litter biomass. Within the saprotrophic guild, the above increase was primarily driven by litter decomposer and wood decaying fungi (mainly basidiomycetes), as opposed to the generalist saprotrophs relying on simple sugars (mainly ascomycetes), as indicated by the relative richness values of the two phyla among the habitats (Supplementary Figure S1).

The soil C content and C/N ratio were lower in VV than in BSC plots, while N content was lower, but not statistically different (Figure 2). Lichen thalli, which make up for a large part of the BSCs biomass, are more easily degradable than woody plants, which could explain the higher soil C content in BSCs.

The greater production of woody litter resistant to decomposition, due to shrub encroachment, especially by *Betula* (Hobbie and Chapin, 1996; Weintraub and Schimel, 2005; Wookey et al., 2009), could result in the reduction of C turnover rates (Natali et al., 2012; Sistla et al., 2013).

We found a positive relationship between the richness of pathogenic fungi and the vegetation cover, possibly due to an increase in the abundance of their hosts and to corresponding changes in water availability and pH values, in accordance with Timling et al. (2014).

Lichenized fungal richness peaked in BSC plots, where lichens are one of the main components of aboveground biomass, with an evident decrease of species richness in VV plots due to competition with vascular plants (Cornelissen et al., 2001; Jägerbrand et al., 2009). Among the edaphic parameters considered, richness of lichenized fungi was negatively correlated to the soil water content, seemed not to be related with pH, and only N content was an independent predictor of community composition, suggesting that other parameters have to be considered for describing the distribution of these species.

Interestingly, not only the increase in shrub coverage, but also its composition, could lead to a deep modification of the Arctic soil ecosystems, as well as C and N fluxes. Changes in vegetation composition influence rhizosphere microbial communities by altering the quality and quantity of litter inputs (Hobbie, 1992; Bardgett, 2005). For example, leaves of *B. nana*, a deciduous shrub strongly encroaching in tundra environments and dominant in the vegetation plots examined, are expected to be decomposed faster than other plant species, given their high nitrogen content (Aerts et al., 2006), but the overall associated litter decomposition may be reduced by the production of a greater proportion of more recalcitrant woody litter (Hobbie and Chapin, 1996; Weintraub and Schimel, 2005; Wookey et al., 2009).

The significant impact of shrub composition on fungal community and on many functional guilds is consistent with well-documented intimate associations between plant species and their mycorrhizal symbionts (Wang and Qiu, 2006) and pathogens (Gilbert and Webb, 2007). Perhaps surprisingly, among the environmental variables considered, the *Salix* percent coverage explained the highest proportion of variance in the composition of animal pathogens. Deciduous shrubs (such as *B. nana* or *S. glauca*) have rapid growth and a high leaf turnover, with little investment in defense. Differently, slow-growing evergreen shrubs (*E. nigrum* and *V. uliginosum*), with slow leaf turnover, invest more in defense (MacLean and Jensen, 1985). Therefore, differences among the preferences of insect species with respect to different shrub species may partly explain the compositional differences in fungal animal pathogens.

## Conclusion

Data presented in this study describe the composition and functionality of fungal communities connected with three different soil environments representing a gradient of vegetation coverage in Western Greenland, never studied before. Overall, our results support the idea that fungal community composition changes in correlation with vegetation coverage among the plots, with concomitant changes in edaphic factors. In particular, we expect a loss of fungal diversity connected with the expansion of shrub vegetation, given the high number of species unique of uncovered plots. We also highlighted some possible interactions among the members of different functional guilds, mainly regarding the degradation of organic matter and nutrient cycling in this nutrient-limited environment. Moreover, considering the soil communities of plots with VV, we were able to discern a strong effect of shrub composition on the spatial distribution of fungal species. These outcomes should be taken into account also in the context of global change and the connected encroachment of shrubs occurring in the Arctic, which could lead to substantial changes in the soil communities, with possible effects on the degradation of the organic matter, as well as C and N fluxes to the atmosphere. In this optic, not only the expansion of VV, but also its composition should be considered, having a determinant effect on associated soil fungal communities and organic matter turnover.

## Data Availability Statement

DNA sequences object of this work have been submitted to NCBI GenBank BioProject PRJNA526618.

## Author Contributions

FC, LZ, and JG planned and designed the experiment, and wrote the manuscript with inputs from LS, SO, and CP. CP collected the samples. FC performed the DNA extraction. FC and JG performed the data processing and analyses.

## Conflict of Interest

The authors declare that the research was conducted in the absence of any commercial or financial relationships that could be construed as a potential conflict of interest.
