# Supplementary material for: Vegetation, pH and Water Content as Main Factors for Shaping Fungal Richness, Community Composition and Functional Guilds Distribution in Soils of Western Greenland
Source: Front Microbiol. 2019 Oct 11;10:2348. doi: 10.3389/fmicb.2019.02348 (PMC6797927; doi:10.3389/fmicb.2019.02348)
Supplement: Supplementary file 1 [file Data_Sheet_1.pdf]

Supplementary Material

1 Supplementary Figures

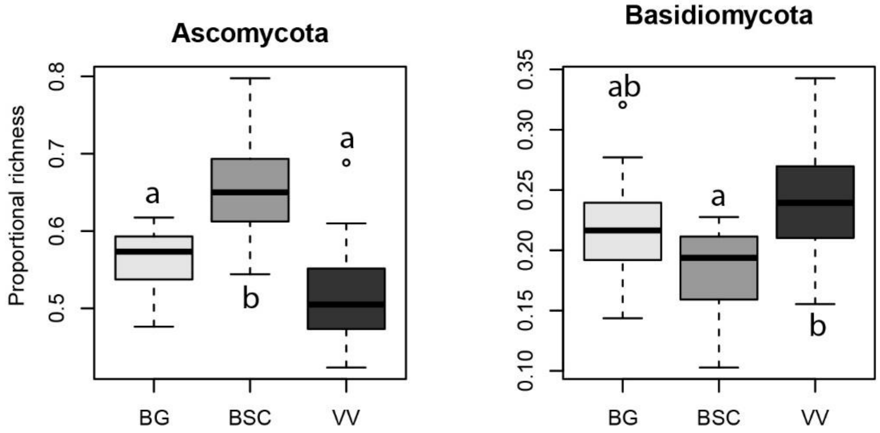

Figure S1: Proportional richness of Ascomycota and Basidiomycota in each habitat (white, Bare Ground plots; light grey, Biological Soil Crusts plots; dark grey, Vascular Vegetation plots). Letters indicate significant differences in one-way ANOVA post-hoc TukeyHSD test (significant for $p<0.05$).

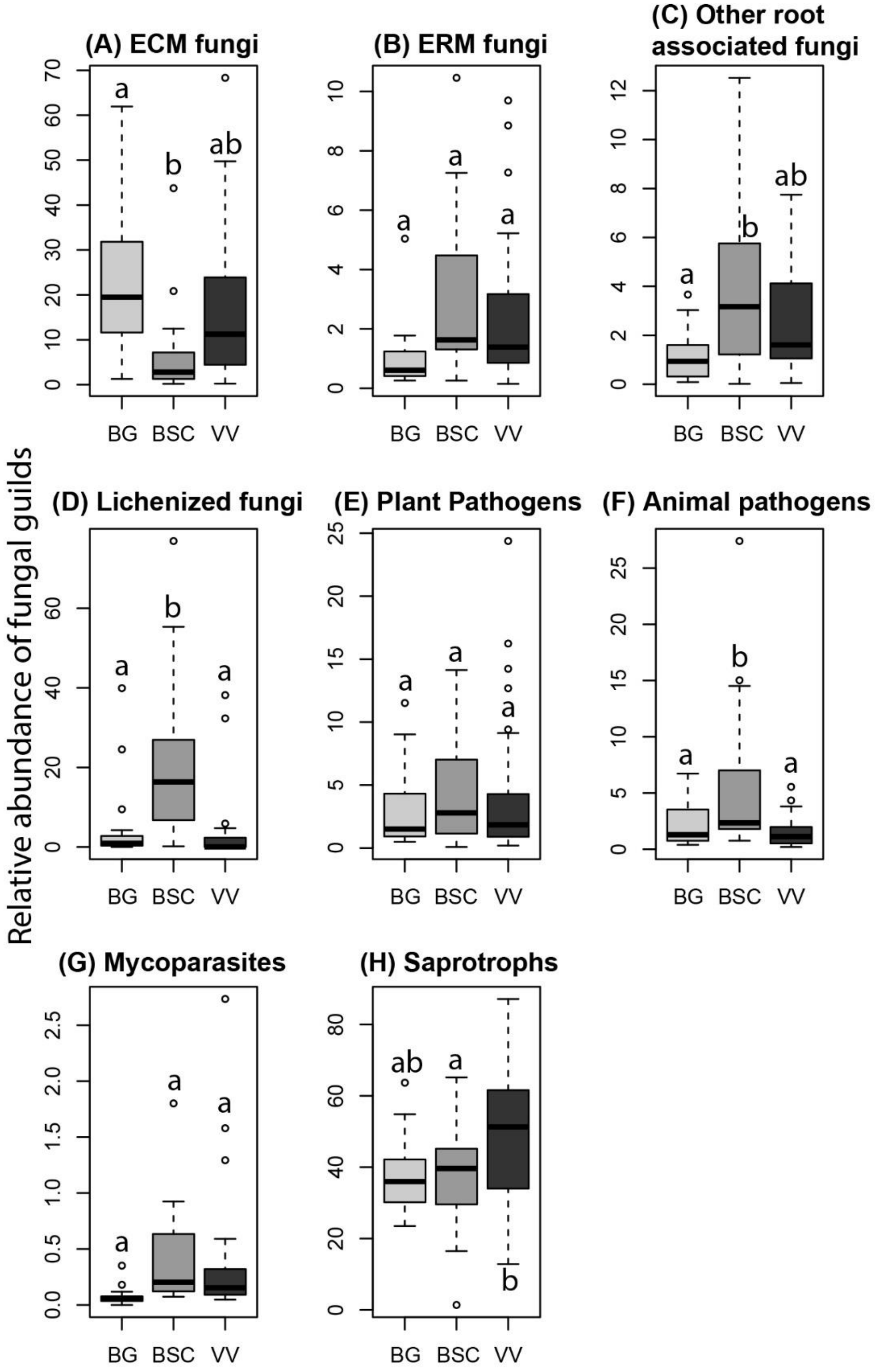

Figure S2 Relative abundance of fungal functional guilds: ectomycorrhizal (ECM), ericoid mycorrhizal (ERM), other root associated, lichenized, plant pathogenic, animal pathogenic, mycoparasites, and saprotrophic fungi in each habitat (light grey, Bare Ground plots; dark grey, Biological Soil Crusts plots; black, Vascular Vegetation plots). Letters indicate significant differences in one-way ANOVA post-hoc TukeyHSD test (significant for $p<0.05$).

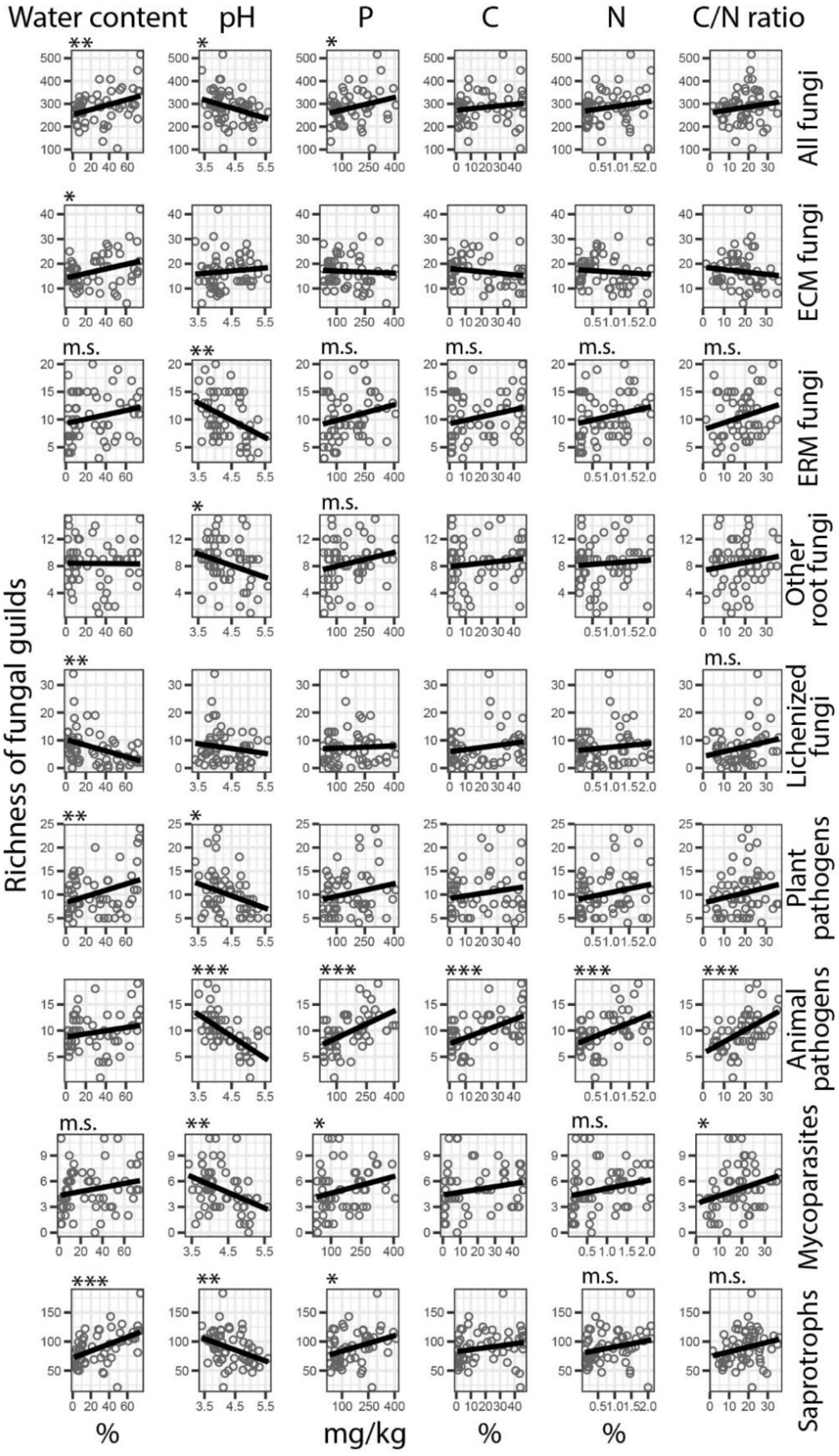

Figure S3: Scatter plots for the variation of richness (y-axis) of the total fungal community and of ectomycorrhizal (ECM), ericoid mycorrhizal (ERM), other root associated, lichenized, mycoparasites, animal pathogens, plant pathogens and saprotrophic fungi, in response to different soil parameters (water content, pH, P, C and N content, and C/N ratio; x-axis). The significance of the regressions is indicated as *** $p<0.001$, ** $p<0.01$, * $p<0.05$, m. s. (marginally significant) $p<0.1$. All the slopes and r^2 values for statistically significant regressions are reported in table S3.

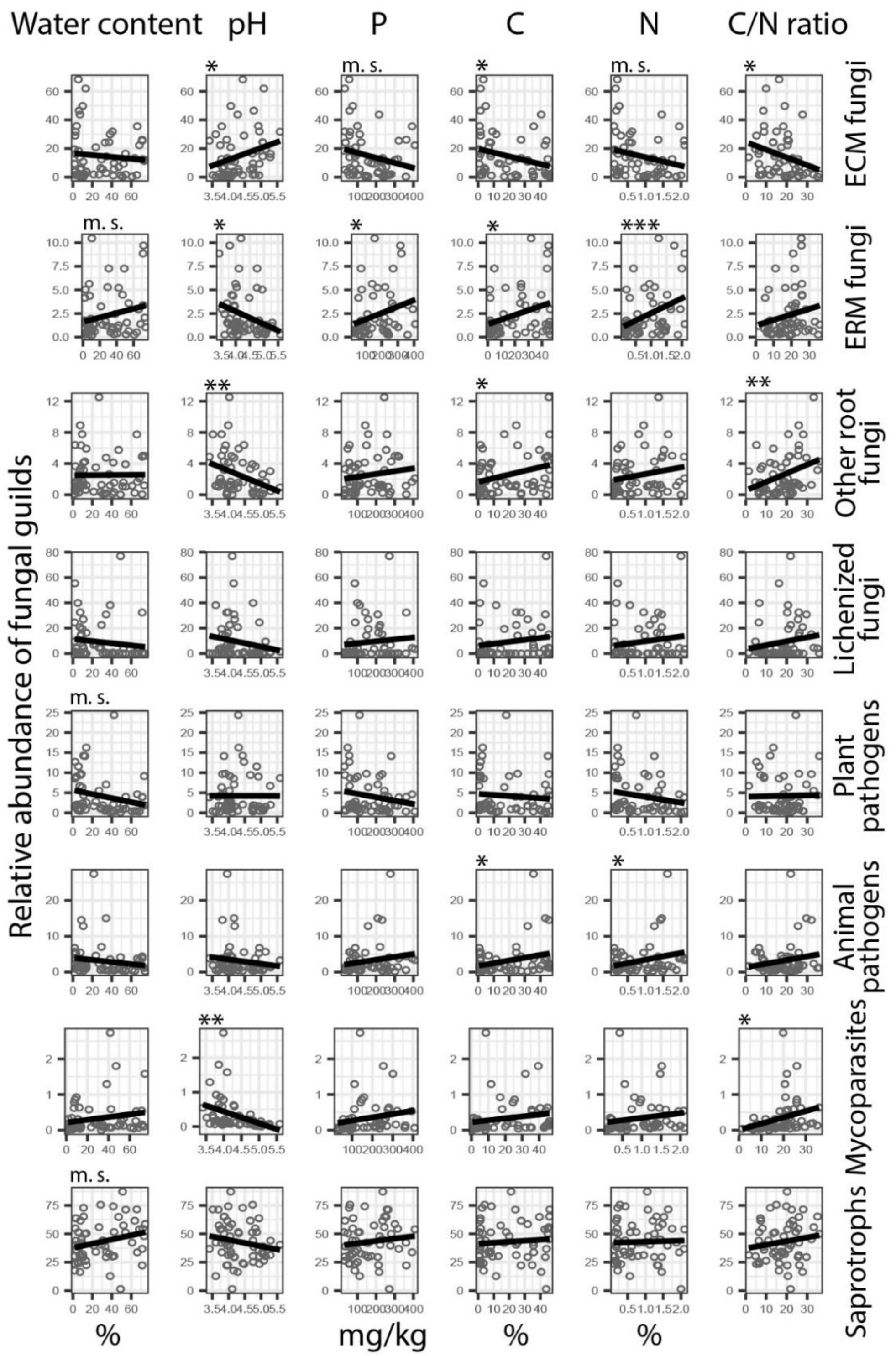

Figure S4 Scatter plots for the variation of relative abundance (y-axis) of ectomycorrhizal (ECM), ericoid mycorrhizal (ERM), other root associated, lichenized, mycoparasites, animal pathogens, plant pathogens and saprotrophic fungi, in response to different soil parameters (water content, pH, P, C and N content, and C/N ratio; x-axis). The significance of the regressions is indicated as *** $p<0.001$, ** $p<0.01$, * $p<0.05$, m. s. (marginally significant) $p<0.1$. All the slopes and r^2 values for statistically significant regressions are reported in table S3.

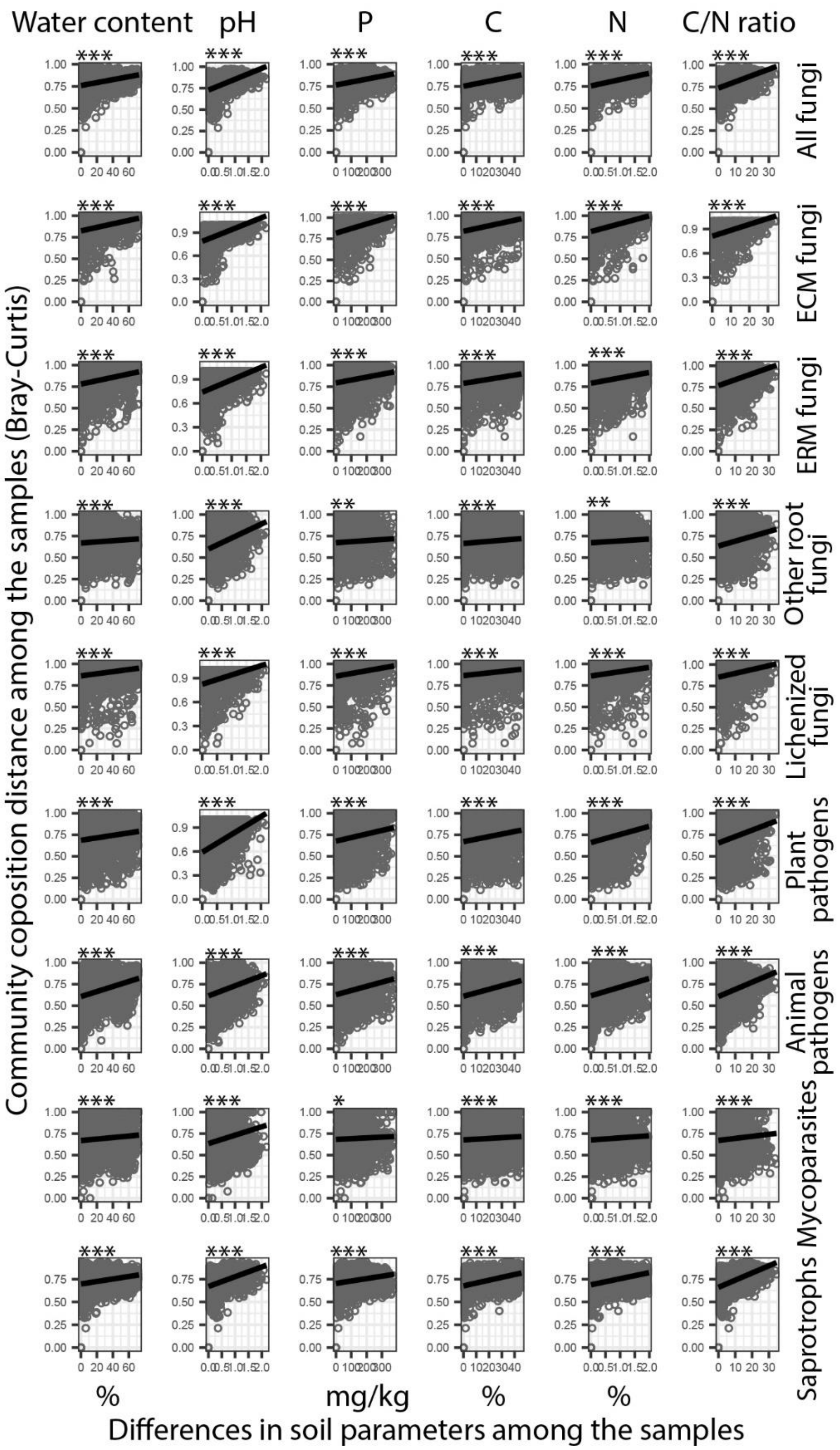

Figure S5: Scatter plots for the differences in community composition (Bray-Curtis distance; y-axis) of the total fungal community and of ectomycorrhizal (ECM), ericoid mycorrhizal (ERM), other root associated, lichenized, mycoparasites, animal pathogens, plant pathogens and saprotrophic fungi, in response to the differences in soil parameters (water content, pH, P, C and N content, and C/N ratio; x-axis) among the samples. The significance of the regressions is indicated as *** $p<0.001$, ** $p<0.01$, * $p<0.05$.

2 Supplementary Tables

Table S1: Characteristics of the 20 sampling plots (altitude and coordinates). For plots of vascular vegetation covered soils the percentage of the most abundant shrub genera are reported

Plots	Altitude (m a.s.l.)	Coordinates	*Salix*	*Betula*	*Vaccinium*	*Empetrum*
Vascular Vegetation plot 1	49	64°07'53"N 51°23'10"W	1	12	5	25
Vascular Vegetation plot 2	23	64°07'55"N 51°23'04"W	0	0	10	60
Vascular Vegetation plot 3	43	64°07'56"N 51°23'02"W	0	90	5	0
Vascular Vegetation plot 4	41	64°07'58"N 51°22'58"W	0	20	35	2
Vascular Vegetation plot 5	34	64°08'04"N 51°22'48"W	0	0	0	60
Vascular Vegetation plot 6	0	64°08'12"N 51°22'36"W	0	62	0	23
Vascular Vegetation plot 7	29	64°08'05"N 51°22'48"W	2	0	15	5
Vascular Vegetation plot 8	68	64°08'18"N 51°22'27"W	0	0	0	90
Vascular Vegetation plot 9	109	64°08'25"N 51°22'18"W	30	0	15	5
Biological Soil Crust plot 1	0	64°08'12"N 51°22'36"W				
Biological Soil Crust plot 2	23	64°07'56"N 51°23'05"W				
Biological Soil Crust plot 3	43	64°07'56"N 51°23'02"W				
Biological Soil Crust plot 4	66	64°07'53"N 51°23'12"W				
Biological Soil Crust plot 5	83	64°07'51"N 51°23'21"W				
Biological Soil Crust plot 6	107	64°07'53"N 51°23'11"W				
Bare Ground plot 1	15	64°14'48"N 51°17'45"W				
Bare Ground plot 2	25	64°10'44"N 51°22'31"W				
Bare Ground plot 3	45	64°07'59"N 51°22'43"W				
Bare Ground plot 4	77	64°07'53"N 51°23'20"W				
Bare Ground plot 5	109	64°08'25"N 51°22'17"W				

Table S2 Fungal OTUs considered as significant indicators of the three habitats (BG: Bare Ground; BSC: Biological Soil Crusts; VV: Vascular Vegetation) with corresponding p-values, assigned functional guilds, and matching Species Hypotheses, ITS1 rDNA sequence similarity (%) of the most similar matching sequence in the UNITE + INSD dynamic Species Hypotheses database

OTU	Habitat	p-value	Match	Functional guild
OTU1399	BG	0,0004	70%|KF297221|SH028591.07FU	
OTU1020	BG	0,0002	70.1%|FJ235869|SH175387.07FU	
OTU1722	BG	0,0004	70.3%|KF618076|SH201685.07FU	
OTU525	BG	0,0154	70.4%|HQ191389|SH011707.07FU	
OTU3271	BG	0,014	70.6%|FN663743|SH105367.07FU	
OTU1753	BG	0,0002	70.6%|KF028767|SH467878.07FU	
OTU757	BG	0,0002	70.6%|KF028767|SH467878.07FU	
OTU252	BG	0,0002	70.6%|KF028767|SH467878.07FU	
OTU516	BG	0,0004	70.6%|KF028767|SH467878.07FU	
OTU522	BG	0,0006	70.6%|KF028767|SH467878.07FU	
OTU904	BG	0,0008	70.6%|KF028767|SH467878.07FU	
OTU1938	BG	0,0022	70.6%|KF028767|SH467878.07FU	
OTU1271	BG	0,0028	70.6%|KF028767|SH467878.07FU	
OTU1148	BG	0,0032	70.6%|KF028767|SH467878.07FU	
OTU2895	BG	0,0044	70.6%|KF028767|SH467878.07FU	
OTU697	BG	0,0128	70.6%|KF028767|SH467878.07FU	
OTU1306	BG	0,0138	70.6%|KF028767|SH467878.07FU	
OTU2104	BG	0,0144	70.6%|KF028767|SH467878.07FU	
OTU2461	BG	0,0156	70.6%|KF028767|SH467878.07FU	
OTU2086	BG	0,0166	70.6%|KF028767|SH467878.07FU	
OTU1277	BG	0,0002	71.1%|JQ003630|SH223176.07FU	
OTU1389	BG	0,0002	71.1%|JQ003630|SH223176.07FU	
OTU1416	BG	0,0002	71.1%|JQ003630|SH223176.07FU	
OTU671	BG	0,0002	71.1%|JQ003630|SH223176.07FU	
OTU686	BG	0,0002	71.1%|JQ003630|SH223176.07FU	
OTU1038	BG	0,0004	71.1%|JQ003630|SH223176.07FU	
OTU2726	BG	0,0004	71.1%|JQ003630|SH223176.07FU	
OTU702	BG	0,0008	71.1%|JQ003630|SH223176.07FU	

OTU413	BG	0,0016	71.1%|JQ003630|SH223176.07FU	
OTU363	BG	0,0018	71.1%|JQ003630|SH223176.07FU	
OTU2195	BG	0,0022	71.1%|JQ003630|SH223176.07FU	
OTU1856	BG	0,0036	71.1%|JQ003630|SH223176.07FU	
OTU1873	BG	0,0036	71.1%|JQ003630|SH223176.07FU	
OTU1759	BG	0,0036	71.1%|JQ003630|SH223176.07FU	
OTU1333	BG	0,0042	71.1%|JQ003630|SH223176.07FU	
OTU1097	BG	0,0048	71.1%|JQ003630|SH223176.07FU	
OTU1492	BG	0,005	71.1%|JQ003630|SH223176.07FU	
OTU619	BG	0,007	71.1%|JQ003630|SH223176.07FU	
OTU2074	BG	0,0116	71.1%|JQ003630|SH223176.07FU	
OTU1593	BG	0,0124	71.1%|JQ003630|SH223176.07FU	
OTU1609	BG	0,0126	71.1%|JQ003630|SH223176.07FU	
OTU1858	BG	0,0128	71.1%|JQ003630|SH223176.07FU	
OTU806	BG	0,0128	71.1%|JQ003630|SH223176.07FU	
OTU1235	BG	0,0132	71.1%|JQ003630|SH223176.07FU	
OTU543	BG	0,0138	71.1%|JQ003630|SH223176.07FU	
OTU2742	BG	0,014	71.1%|JQ003630|SH223176.07FU	
OTU2975	BG	0,0156	71.1%|JQ003630|SH223176.07FU	
OTU1252	BG	0,0218	71.1%|JQ003630|SH223176.07FU	
OTU1322	BG	0,0278	71.1%|JQ003630|SH223176.07FU	
OTU979	BG	0,0002	71.4%|HQ191389|SH011707.07FU	
OTU953	BG	0,0026	71.5%|KC965904|SH466422.07FU	
OTU981	BG	0,0028	71.6%|KF296986|SH215255.07FU	
OTU505	BG	0,0238	71.8%|KP889858|SH491074.07FU	
OTU1197	BG	0,0002	72%|HQ191389|SH011707.07FU	
OTU195	BG	0,0004	72.1%|JF300543|SH462390.07FU	
OTU1422	BG	0,0022	72.5%|JF300413|SH467423.07FU	
OTU541	BG	0,0018	72.5%|KF028767|SH467878.07FU	
OTU2019	BG	0,0026	72.5%|KF028767|SH467878.07FU	
OTU1532	BG	0,0216	72.6%|KP889469|SH488280.07FU	

OTU1519	BG	0,0134	72.7%|KF618076|SH201685.07FU	
OTU1147	BG	0,0132	72.8%|HQ191389|SH011707.07FU	
OTU1054	BG	0,0128	73%|JF300543|SH462390.07FU	
OTU1954	BG	0,025	73.2%|KC966225|SH479904.07FU	
OTU630	BG	0,0068	73.4%|FJ903326|SH209339.07FU	
OTU936	BG	0,02	73.6%|EU167587|SH016560.07FU	
OTU1604	BG	0,0052	73.6%|LC095210|SH214466.07FU	
OTU1237	BG	0,0002	73.7%|FN610984|SH476131.07FU	
OTU556	BG	0,0238	73.7%|JX384329|SH534410.07FU	
OTU339	BG	0,0024	73.8%|DQ421206|SH180898.07FU	
OTU576	BG	0,0008	74%|KF296953|SH485511.07FU	
OTU1623	BG	0,001	74.5%|KF028767|SH467878.07FU	
OTU2738	BG	0,019	74.5%|KF618164|SH482592.07FU	
OTU2267	BG	0,014	74.6%|KJ462332|SH494064.07FU	
OTU1374	BG	0,0152	75.5%|HQ191359|SH192017.07FU	
OTU1692	BG	0,0152	75.5%|KF617838|SH459212.07FU	
OTU471	BG	0,0002	76.4%|UDB014680|SH489219.07FU	
OTU2433	BG	0,0004	76.7%|FJ554329|SH208595.07FU	
OTU1420	BG	0,0248	76.7%|KF573614|SH020574.07FU	
OTU1797	BG	0,028	77%|KR857021|SH521434.07FU	
OTU190	BG	0,0026	77.2%|KC965545|SH027987.07FU	
OTU1581	BG	0,0006	77.2%|KM371464|SH533907.07FU	
OTU1184	BG	0,0012	77.2%|KT160787|SH491269.07FU	
OTU917	BG	0,0176	77.6%|KF297043|SH466107.07FU	
OTU249	BG	0,0002	77.8%|JX036117|SH209262.07FU	
OTU1485	BG	0,0012	78.2%|KU862933|SH631204.07FU	
OTU269	BG	0,0002	78.7%|FJ554329|SH208595.07FU	
OTU2101	BG	0,0002	78.7%|KF297056|SH019692.07FU	
OTU586	BG	0,0152	78.8%|KT378114|SH489500.07FU	
OTU1124	BG	0,001	78.8%|KU582498|SH524414.07FU	
OTU121	BG	0,0008	79.2%|HQ211500|SH185974.07FU	
OTU50	BG	0,0002	79.3%|KC965280|SH019693.07FU	

OTU66	BG	0,0008	79.3%|KM371464|SH533907.07FU	
OTU1910	BG	0,014	79.4%|KF297043|SH466107.07FU	
OTU49	BG	0,0002	80.3%|KC965545|SH027987.07FU	
OTU2594	BG	0,0002	80.7%|FJ554329|SH208595.07FU	
OTU996	BG	0,0152	81%|KC966183|SH469075.07FU	
OTU579	BG	0,0152	81%|KC966183|SH469075.07FU	
OTU1607	BG	0,0004	81.1%|KP889623|SH493699.07FU	
OTU106	BG	0,0004	81.7%|KC965145|SH029989.07FU	
OTU1435	BG	0,0156	82.2%|JQ666573|SH022011.07FU	
OTU598	BG	0,0056	82.3%|JN040508|SH186600.07FU	
OTU616	BG	0,0004	82.8%|KF617751|SH004874.07FU	
OTU259	BG	0,0004	83%|KF296938|SH463566.07FU	
OTU1293	BG	0,0128	83.5%|KP889609|SH496492.07FU	
OTU1741	BG	0,0128	83.5%|KP889680|SH488273.07FU	
OTU1362	BG	0,017	83.8%|KF227866|SH186962.07FU	
OTU1923	BG	0,0032	83.9%|KU582212|SH492309.07FU	
OTU547	BG	0,037	84.5%|JQ666721|SH027463.07FU	
OTU1218	BG	0,0292	84.9%|FJ645262|SH462404.07FU	
OTU2371	BG	0,0152	85.3%|KF296918|SH474311.07FU	
OTU2776	BG	0,0128	85.8%|AB968620|SH215930.07FU	
OTU178	BG	0,0028	85.9%|FJ553008|SH215546.07FU	
OTU2502	BG	0,0138	85.9%|HQ211814|SH000254.07FU	
OTU840	BG	0,0126	86.1%|JX489782|SH020438.07FU	
OTU408	BG	0,0138	86.5%|HM161474|SH192355.07FU	
OTU531	BG	0,0292	87.1%|HQ211499|SH221757.07FU	
OTU242	BG	0,0006	87.9%|KC965974|SH459702.07FU	
OTU388	BG	0,0002	88%|KC965545|SH027987.07FU	
OTU168	BG	0,0226	88%|KF937321|SH493044.07FU	
OTU928	BG	0,0008	88.4%|GU256952|SH019741.07FU	
OTU428	BG	0,0192	88.7%|KC965974|SH459702.07FU	
OTU1633	BG	0,0138	89.4%|FN610873|SH204500.07FU	

OTU736	BG	0,0236	89.6%|KF617747|SH204499.07FU	
OTU375	BG	0,0192	89.7%|GU328580|SH004147.07FU	
OTU374	BG	0,0004	89.7%|KP756520|SH629666.07FU	
OTU110	BG	0,0012	89.9%|DQ822815|SH214854.07FU	
OTU2924	BG	0,0142	89.9%|KC965434|SH004626.07FU	
OTU677	BG	0,0004	93%|HQ829336|SH204501.07FU	
OTU217	BG	0,0002	93%|KC965545|SH027987.07FU	
OTU500	BG	0,0028	94%|HQ211503|SH031257.07FU	
OTU292	BG	0,0126	95%|HQ211607|SH201713.07FU	
OTU113	BG	0,014	95%|JQ666570|SH214618.07FU	
OTU929	BG	0,019	97%|JN032561|SH209947.07FU	
OTU1810	BG	0,0252	97%|KU176320|SH184453.07FU	
OTU424	BG	0,0024	98%|KF617659|SH203524.07FU	
OTU626	BG	0,0264	99%|LC131027|SH631398.07FU	
OTU1285	BG	0,0464	100%|JN885548|SH200713.07FU	Saprotroph
OTU473	BG	0,014	96.8%|JQ346870|SH181489.07FU	Animal Pathogen
OTU114	BG	0,0062	97.6%|FJ554329|SH208595.07FU	Animal Pathogen
OTU577	BG	0,0152	93.9%|KP756480|SH640122.07FU	Arbuscular Mycorrhizal
OTU2355	BG	0,0152	94.9%|KP756480|SH640122.07FU	Arbuscular Mycorrhizal
OTU443	BG	0,0004	96.1%|DQ400126|SH005209.07FU	Arbuscular Mycorrhizal
OTU596	BG	0,0152	96.4%|KP756480|SH640122.07FU	Arbuscular Mycorrhizal
OTU866	BG	0,0046	96.6%|KF206544|SH203297.07FU	Arbuscular Mycorrhizal
OTU599	BG	0,0152	96.9%|KP756480|SH640122.07FU	Arbuscular Mycorrhizal
OTU649	BG	0,001	98.6%|HE858428|SH194637.07FU	Arbuscular Mycorrhizal
OTU316	BG	0,0112	98.9%|FJ554264|SH011124.07FU	Arbuscular Mycorrhizal
OTU307	BG	0,001	100%|DQ102671|SH188476.07FU	Ectomycorrhizal
OTU152	BG	0,0006	100%|JN580877|SH214853.07FU	Ectomycorrhizal
OTU34	BG	0,0002	100%|JQ711860|SH188594.07FU	Ectomycorrhizal
OTU1136	BG	0,0052	93.7%|AF083205|SH014937.07FU	Ectomycorrhizal
OTU115	BG	0,0002	94%|AY534209|SH187320.07FU	Ectomycorrhizal
OTU2397	BG	0,0002	95.8%|KF617237|SH181110.07FU	Ectomycorrhizal
OTU174	BG	0,0002	96.3%|KF617717|SH201237.07FU	Ectomycorrhizal

OTU965	BG	0,0152	96.4%|UDB021463|SH135196.07FU	Ectomycorrhizal
OTU8	BG	0,0006	96.6%|AB354285|SH030455.07FU	Ectomycorrhizal
OTU302	BG	0,014	97.3%|AM882947|SH188901.07FU	Ectomycorrhizal
OTU62	BG	0,0128	97.8%|UDB012704|SH184885.07FU	Ectomycorrhizal
OTU51	BG	0,0002	98.3%|KF617717|SH201237.07FU	Ectomycorrhizal
OTU39	BG	0,0004	98.7%|KF007250|SH210197.07FU	Ectomycorrhizal
OTU1351	BG	0,0128	99.2%|FM992957|SH219260.07FU	Ectomycorrhizal
OTU1325	BG	0,0192	99.2%|GU234030|SH190354.07FU	Ectomycorrhizal
OTU314	BG	0,0128	99.2%|HM488519|SH197132.07FU	Ectomycorrhizal
OTU134	BG	0,0066	99.2%|HQ211814|SH000254.07FU	Ectomycorrhizal
OTU180	BG	0,0494	99.3%|HM164617|SH177806.07FU	Ectomycorrhizal
OTU1599	BG	0,0002	99.6%|HQ212304|SH174317.07FU	Ectomycorrhizal
OTU353	BG	0,0006	99.6%|JQ003657|SH218066.07FU	Ectomycorrhizal
OTU1863	BG	0,0066	100%|KY522966|SH628928.07FU	Ericoid mycorrhizal
OTU2406	BG	0,0254	93.5%|FN565259|SH204995.07FU	Ericoid mycorrhizal
OTU364	BG	0,0058	96.5%|JX317229|SH211410.07FU	Ericoid mycorrhizal
OTU254	BG	0,0006	97.6%|KF617746|SH205746.07FU	Ericoid mycorrhizal
OTU1279	BG	0,0128	98.4%|GQ223468|SH007166.07FU	Ericoid mycorrhizal
OTU882	BG	0,0086	98.6%|FM997952|SH012946.07FU	Ericoid mycorrhizal
OTU1041	BG	0,0032	95.6%|HQ211717|SH193767.07FU	Fungal parasite
OTU310	BG	0,0044	100%|KJ462265|SH473594.07FU	Lichenized
OTU1617	BG	0,014	94%|KM243179|SH533246.07FU	Lichenized
OTU568	BG	0,0002	94.7%|AY756479|SH016365.07FU	Lichenized
OTU1134	BG	0,014	96.7%|KM243179|SH533246.07FU	Lichenized
OTU40	BG	0,0004	97.4%|HQ446017|SH027985.07FU	Lichenized
OTU2180	BG	0,0012	97.8%|KJ162312|SH210524.07FU	Lichenized
OTU1165	BG	0,0002	98%|AY756479|SH016365.07FU	Lichenized
OTU199	BG	0,0016	98.1%|KJ162312|SH210524.07FU	Lichenized
OTU2412	BG	0,0024	99.6%|GU074430|SH210507.07FU	Lichenized
OTU13	BG	0,0004	99.6%|HQ446017|SH027985.07FU	Lichenized
OTU11	BG	0,0002	96.7%|AJ878779|SH016893.07FU	Litter saprotrophUndefined saprotroph

OTU287	BG	0,0002	99%|HQ630348|SH187868.07FU	Litter saprotrophUndefined saprotroph
OTU33	BG	0,0002	99.6%|HQ021782|SH191111.07FU	Litter saprotrophUndefined saprotroph
OTU234	BG	0,003	100%|HQ873705|SH183987.07FU	Plant Pathogen
OTU136	BG	0,0002	93.7%|KM359781|SH523879.07FU	Plant Pathogen
OTU1112	BG	0,0146	96.3%|AB968620|SH215930.07FU	Plant Pathogen
OTU279	BG	0,0264	96.3%|JX857179|SH218295.07FU	Plant Pathogen
OTU1973	BG	0,0282	93.8%|KC965434|SH004626.07FU	Plant saprotrophWood saprotroph
OTU2602	BG	0,0002	98.9%|KC965434|SH004626.07FU	Plant saprotrophWood saprotroph
OTU931	BG	0,0044	100%|UDB028015|SH494690.07FU	Root associated fungus
OTU493	BG	0,0128	99.6%|AJ633124|SH187930.07FU	Root associated fungus
OTU817	BG	0,004	100%|AF062818|SH183330.07FU	Undefined saprotroph
OTU191	BG	0,0002	100%|AY443470|SH407688.07FU	Undefined saprotroph
OTU9	BG	0,0002	100%|DQ069044|SH204986.07FU	Undefined saprotroph
OTU216	BG	0,0074	100%|DQ315441|SH181342.07FU	Undefined saprotroph
OTU103	BG	0,0024	100%|FJ378855|SH201605.07FU	Undefined saprotroph
OTU87	BG	0,0024	100%|GQ411337|SH202969.07FU	Undefined saprotroph
OTU754	BG	0,0012	100%|HG935790|SH175136.07FU	Undefined saprotroph
OTU240	BG	0,0002	100%|JF300706|SH190020.07FU	Undefined saprotroph
OTU299	BG	0,0262	100%|JN569102|SH184430.07FU	Undefined saprotroph
OTU593	BG	0,0292	100%|JQ256426|SH174885.07FU	Undefined saprotroph
OTU1102	BG	0,0274	100%|JQ668731|SH180668.07FU	Undefined saprotroph
OTU48	BG	0,0004	100%|JX043061|SH029391.07FU	Undefined saprotroph
OTU715	BG	0,0042	100%|LC177643|SH208860.07FU	Undefined saprotroph
OTU332	BG	0,0128	91.6%|EF433966|SH029887.07FU	Undefined saprotroph
OTU459	BG	0,0004	91.6%|KF617637|SH208638.07FU	Undefined saprotroph
OTU1626	BG	0,0014	92.1%|AY781244|SH028081.07FU	Undefined saprotroph
OTU278	BG	0,014	92.8%|HQ154328|SH003907.07FU	Undefined saprotroph
OTU127	BG	0,0138	93.1%|KF617750|SH004446.07FU	Undefined saprotroph
OTU1533	BG	0,0002	93.3%|LC096595|SH630111.07FU	Undefined saprotroph
OTU465	BG	0,0008	93.6%|KC965981|SH028079.07FU	Undefined saprotroph
OTU377	BG	0,0132	93.8%|KT160948|SH497117.07FU	Undefined saprotroph
OTU688	BG	0,016	94.8%|EU917116|SH014306.07FU	Undefined saprotroph

OTU1185	BG	0,0334	95.2%|GU174395|SH014257.07FU	Undefined saprotroph
OTU1052	BG	0,0498	95.3%|KC965665|SH018520.07FU	Undefined saprotroph
OTU3284	BG	0,0042	96.2%|EF434088|SH188392.07FU	Undefined saprotroph
OTU1220	BG	0,001	96.2%|HM036653|SH180664.07FU	Undefined saprotroph
OTU1356	BG	0,0024	96.6%|KC489485|SH216098.07FU	Undefined saprotroph
OTU410	BG	0,007	97.4%|FJ553969|SH209863.07FU	Undefined saprotroph
OTU653	BG	0,022	97.5%|JN899387|SH200696.07FU	Undefined saprotroph
OTU607	BG	0,0104	97.5%|KM246506|SH497060.07FU	Undefined saprotroph
OTU1000	BG	0,0086	97.6%|JX270418|SH216995.07FU	Undefined saprotroph
OTU255	BG	0,0366	97.6%|KM504411|SH217025.07FU	Undefined saprotroph
OTU643	BG	0,0006	98.1%|KF617609|SH479309.07FU	Undefined saprotroph
OTU488	BG	0,0002	98.3%|HQ211698|SH181990.07FU	Undefined saprotroph
OTU913	BG	0,0002	98.3%|KM246506|SH497060.07FU	Undefined saprotroph
OTU369	BG	0,0152	98.5%|GU174419|SH025553.07FU	Undefined saprotroph
OTU88	BG	0,0002	99%|JN995638|SH198656.07FU	Undefined saprotroph
OTU636	BG	0,0002	99.1%|KT160948|SH497117.07FU	Undefined saprotroph
OTU73	BG	0,003	99.2%|GQ120990|SH022294.07FU	Undefined saprotroph
OTU308	BG	0,0078	99.2%|HM240166|SH208301.07FU	Undefined saprotroph
OTU164	BG	0,002	99.2%|UDB014919|SH207730.07FU	Undefined saprotroph
OTU770	BG	0,015	99.5%|KC019887|SH028064.07FU	Undefined saprotroph
OTU83	BG	0,0008	99.5%|KY782284|SH220112.07FU	Undefined saprotroph
OTU491	BG	0,0028	90%|JF439140|SH005211.07FU	Vesicular-arbuscular mycorrhizal
OTU260	BG	0,0026	98.3%|KF618031|SH212624.07FU	Wood saprotroph
OTU1449	BG	0,0036	90.4%|EF434104|SH183302.07FU	
OTU1535	BG	0,0152	91.1%|KC965903|SH459100.07FU	
OTU2531	BG	0,0138	91.3%|KC965545|SH027987.07FU	
OTU335	BG	0,0016	91.4%|KC965545|SH027987.07FU	
OTU851	BG	0,0028	91.7%|KF618198|SH474570.07FU	
OTU502	BG	0,0144	92.2%|KF274396|SH019990.07FU	
OTU535	BG	0,0002	92.8%|HQ212060|SH218288.07FU	
OTU342	BG	0,0002	93.6%|KF617532|SH473856.07FU	

OTU737	BG	0,0014	93.8%|KF617587|SH215245.07FU	
OTU147	BG	0,0004	94.2%|HF947860|SH218294.07FU	
OTU781	BG	0,0138	94.3%|KC965284|SH464721.07FU	
OTU475	BG	0,0002	94.4%|KF617532|SH473856.07FU	
OTU573	BG	0,0002	94.4%|KF617532|SH473856.07FU	
OTU2154	BG	0,0128	94.5%|EU292461|SH218298.07FU	
OTU519	BG	0,0002	94.8%|KF617532|SH473856.07FU	
OTU805	BG	0,0016	95.6%|EF521253|SH004454.07FU	
OTU67	BG	0,0002	95.8%|KX609411|SH218810.07FU	
OTU538	BG	0,0002	95.9%|KF274302|SH020327.07FU	
OTU2936	BG	0,0022	96.4%|KC965549|SH023667.07FU	
OTU317	BG	0,0002	96.4%|KF617532|SH473856.07FU	
OTU3433	BG	0,0246	96.6%|KF617864|SH220712.07FU	
OTU631	BG	0,0026	98.4%|DQ421206|SH180898.07FU	
OTU346	BG	0,0128	98.8%|KU176283|SH497104.07FU	
OTU1894	BG	0,0288	99.1%|KF617751|SH004874.07FU	
OTU224	BG	0,0002	99.3%|FJ626929|SH460857.07FU	
OTU59	BG	0,0148	99.6%|KF617590|SH212904.07FU	
OTU1259	BSC	0,0004	70.6%|KF028767|SH467878.07FU	
OTU2835	BSC	0,0028	70.6%|KF028767|SH467878.07FU	
OTU1108	BSC	0,005	70.6%|KF028767|SH467878.07FU	
OTU2829	BSC	0,0082	70.6%|KF028767|SH467878.07FU	
OTU1751	BSC	0,0082	70.6%|KF028767|SH467878.07FU	
OTU1364	BSC	0,0142	70.6%|KF028767|SH467878.07FU	
OTU2243	BSC	0,0358	70.6%|KF028767|SH467878.07FU	
OTU1600	BSC	0,0372	70.6%|KF028767|SH467878.07FU	
OTU2207	BSC	0,0112	71.1%|JQ003630|SH223176.07FU	
OTU990	BSC	0,0146	71.1%|JQ003630|SH223176.07FU	
OTU1791	BSC	0,034	71.1%|JQ003630|SH223176.07FU	
OTU511	BSC	0,0004	71.4%|GU328524|SH015441.07FU	
OTU768	BSC	0,001	71.4%|JX109860|SH198216.07FU	
OTU433	BSC	0,02	71.8%|AM882963|SH200258.07FU	

OTU1454	BSC	0,0328	71.9%|FJ554329|SH208595.07FU	
OTU2264	BSC	0,036	72.1%|KF274449|SH474038.07FU	
OTU1404	BSC	0,026	72.1%|KP889572|SH492688.07FU	
OTU451	BSC	0,0002	72.5%|KF028767|SH467878.07FU	
OTU1040	BSC	0,0078	72.5%|KF028767|SH467878.07FU	
OTU477	BSC	0,0008	72.5%|KT328789|SH494084.07FU	
OTU1783	BSC	0,0014	73%|KC965534|SH486771.07FU	
OTU3444	BSC	0,0356	73.1%|HM240125|SH199186.07FU	
OTU427	BSC	0,0024	73.4%|FJ554329|SH208595.07FU	
OTU2219	BSC	0,0234	73.4%|FN868596|SH210044.07FU	
OTU1073	BSC	0,0078	73.5%|LT798821|SH630266.07FU	
OTU1164	BSC	0,0002	73.6%|GU721966|SH208425.07FU	
OTU1758	BSC	0,002	73.8%|KF617838|SH459212.07FU	
OTU3239	BSC	0,037	74%|KF614863|SH213331.07FU	
OTU1710	BSC	0,0086	74.5%|KF028767|SH467878.07FU	
OTU1903	BSC	0,0002	74.8%|EF016381|SH193236.07FU	
OTU108	BSC	0,0002	74.8%|KP889438|SH488681.07FU	
OTU910	BSC	0,0004	75.1%|HQ211717|SH193767.07FU	
OTU613	BSC	0,0024	75.1%|KF617838|SH459212.07FU	
OTU1819	BSC	0,0012	75.3%|KF297043|SH466107.07FU	
OTU347	BSC	0,0002	75.5%|KU141266|SH494194.07FU	
OTU210	BSC	0,0002	75.6%|KP889649|SH495466.07FU	
OTU232	BSC	0,0102	75.9%|KC965874|SH201099.07FU	
OTU1104	BSC	0,0302	76.4%|KT328828|SH491141.07FU	
OTU746	BSC	0,0214	76.4%|KX273438|SH640165.07FU	
OTU985	BSC	0,0018	76.7%|KF297043|SH466107.07FU	
OTU366	BSC	0,0026	77.2%|DQ248314|SH021199.07FU	
OTU1234	BSC	0,0004	77.5%|HQ211717|SH193767.07FU	
OTU684	BSC	0,0026	77.6%|JX857221|SH203320.07FU	
OTU406	BSC	0,0004	77.7%|FJ553822|SH470720.07FU	
OTU897	BSC	0,0004	77.7%|HQ211717|SH193767.07FU	

OTU1349	BSC	0,0002	77.8%|FJ553238|SH193764.07FU	
OTU1012	BSC	0,0284	77.9%|KU863000|SH205887.07FU	
OTU3161	BSC	0,009	78%|UDB024206|SH210017.07FU	
OTU352	BSC	0,0032	78.1%|FJ554329|SH208595.07FU	
OTU1151	BSC	0,0004	78.5%|GU721966|SH208425.07FU	
OTU1015	BSC	0,0008	78.8%|KF617838|SH459212.07FU	
OTU273	BSC	0,0002	79%|HQ211717|SH193767.07FU	
OTU1245	BSC	0,0096	79%|JF300564|SH012950.07FU	
OTU1026	BSC	0,033	80%|FJ553396|SH208590.07FU	
OTU1531	BSC	0,0082	80%|KF274277|SH025617.07FU	
OTU923	BSC	0,0002	80.8%|AM901817|SH023176.07FU	
OTU214	BSC	0,0002	80.9%|DQ054542|SH005081.07FU	
OTU812	BSC	0,044	81.6%|JQ768940|SH215449.07FU	
OTU2687	BSC	0,0078	81.9%|KP889623|SH493699.07FU	
OTU3159	BSC	0,0018	82.5%|KC965900|SH183192.07FU	
OTU1834	BSC	0,0052	82.5%|KM246219|SH495771.07FU	
OTU1403	BSC	0,0104	83.5%|AM260819|SH208594.07FU	
OTU228	BSC	0,0188	83.9%|HQ211499|SH221757.07FU	
OTU455	BSC	0,0202	84.1%|KP889390|SH496500.07FU	
OTU294	BSC	0,0002	84.2%|KC965900|SH183192.07FU	
OTU1426	BSC	0,0002	84.7%|AM260819|SH208594.07FU	
OTU248	BSC	0,0042	84.8%|KF618021|SH023175.07FU	
OTU1198	BSC	0,0168	84.9%|FJ827709|SH210200.07FU	
OTU1200	BSC	0,0026	84.9%|KJ462279|SH527325.07FU	
OTU2547	BSC	0,0342	85%|JN863281|SH210018.07FU	
OTU708	BSC	0,0004	85%|KC965361|SH025708.07FU	
OTU758	BSC	0,0004	85.4%|KC884701|SH027203.07FU	
OTU1456	BSC	0,0364	85.7%|KF274410|SH014522.07FU	
OTU404	BSC	0,0004	85.7%|KP986517|SH496367.07FU	
OTU1095	BSC	0,0328	86.5%|JN863281|SH210018.07FU	
OTU1438	BSC	0,0484	86.6%|KC966354|SH023345.07FU	
OTU681	BSC	0,0228	86.7%|HQ212020|SH182087.07FU	

OTU665	BSC	0,0048	86.8%|KJ462276|SH527328.07FU	
OTU776	BSC	0,0358	87.1%|KP889526|SH204558.07FU	
OTU226	BSC	0,0066	87.2%|KP889390|SH496500.07FU	
OTU450	BSC	0,0436	87.8%|GU187531|SH472365.07FU	
OTU2310	BSC	0,0014	87.9%|FJ013066|SH184510.07FU	
OTU594	BSC	0,0002	88.4%|FJ008692|SH023343.07FU	
OTU367	BSC	0,0002	88.8%|JN032542|SH183189.07FU	
OTU499	BSC	0,0004	88.8%|KP889976|SH496541.07FU	
OTU1236	BSC	0,0328	89.1%|JX857179|SH218295.07FU	
OTU701	BSC	0,0014	89.8%|FN565212|SH217841.07FU	
OTU266	BSC	0,0058	90%|EF373563|SH005236.07FU	
OTU1013	BSC	0,004	90%|KC966354|SH023345.07FU	
OTU818	BSC	0,0032	96%|KC965669|SH027346.07FU	
OTU321	BSC	0,0002	98%|AM260819|SH208594.07FU	
OTU1513	BSC	0,0062	99%|AM999674|SH205434.07FU	
OTU1516	BSC	0,0456	99%|KF274414|SH020335.07FU	
OTU19	BSC	0,0002	100%|FM997929|SH208599.07FU	Animal Pathogen
OTU1465	BSC	0,0118	100%|KF297148|SH193260.07FU	Animal Pathogen
OTU2470	BSC	0,0342	92.4%|HQ267956|SH029166.07FU	Animal Pathogen
OTU1265	BSC	0,0056	92.5%|FN555433|SH012598.07FU	Animal Pathogen
OTU157	BSC	0,0002	95.7%|FJ554329|SH208595.07FU	Animal Pathogen
OTU167	BSC	0,0024	96.3%|KF225793|SH474367.07FU	Animal pathogen
OTU85	BSC	0,0002	97.6%|FJ554329|SH208595.07FU	Animal Pathogen
OTU1250	BSC	0,0464	100%|HE605213|SH199199.07FU	Animal PathogenUndefined saprotroph
OTU970	BSC	0,0048	96.3%|JX984761|SH205856.07FU	Dung saprotrophPlant saprotroph
OTU1206	BSC	0,0008	99.2%|KC254096|SH253710.07FU	Dung saprotrophWood saprotroph
OTU510	BSC	0,0002	100%|FJ475663|SH214269.07FU	Ectomycorrhizal
OTU2177	BSC	0,0252	100%|KM504406|SH203693.07FU	Ectomycorrhizal
OTU504	BSC	0,0404	100%|KX423754|SH631462.07FU	Ectomycorrhizal
OTU407	BSC	0,0416	100%|KY522914|SH630271.07FU	Ectomycorrhizal
OTU10	BSC	0,0228	97.6%|FJ013066|SH184510.07FU	Ectomycorrhizal

OTU38	BSC	0,0002	98.4%|FJ475673|SH461592.07FU	Ectomycorrhizal
OTU1006	BSC	0,0184	100%|AB476488|SH208582.07FU	Ericoid mycorrhizal
OTU546	BSC	0,0002	90.1%|HQ260285|SH014523.07FU	Ericoid mycorrhizal
OTU3251	BSC	0,0082	92.1%|AM260819|SH208594.07FU	Ericoid mycorrhizal
OTU3305	BSC	0,0348	92.1%|AM260819|SH208594.07FU	Ericoid mycorrhizal
OTU464	BSC	0,0008	95.5%|DQ309140|SH019516.07FU	Ericoid mycorrhizal
OTU3219	BSC	0,0002	96.8%|AM260819|SH208594.07FU	Ericoid mycorrhizal
OTU139	BSC	0,0002	98.4%|AM260819|SH208594.07FU	Ericoid mycorrhizal
OTU45	BSC	0,0172	99.6%|KC966311|SH201642.07FU	Ericoid mycorrhizal
OTU992	BSC	0,0374	100%|KF274145|SH025132.07FU	Fungal Parasite
OTU3419	BSC	0,0006	97.4%|KF274139|SH013369.07FU	Fungal Parasite
OTU783	BSC	0,0028	98.9%|HQ211619|SH182180.07FU	Fungal Parasite
OTU2952	BSC	0,0074	90.5%|JN863281|SH210018.07FU	Lichenized
OTU1694	BSC	0,0324	91%|FM205913|SH212241.07FU	Lichenized
OTU15	BSC	0,0002	91.2%|JN863281|SH210018.07FU	Lichenized
OTU2875	BSC	0,0014	91.5%|FN868596|SH210044.07FU	Lichenized
OTU1191	BSC	0,0002	92.1%|FM205913|SH212241.07FU	Lichenized
OTU1065	BSC	0,0002	92.4%|KC965805|SH005235.07FU	Lichenized
OTU532	BSC	0,0016	92.5%|JN863281|SH210018.07FU	Lichenized
OTU1505	BSC	0,0002	93.1%|UDB024202|SH210064.07FU	Lichenized
OTU137	BSC	0,0018	93.4%|FN868596|SH210044.07FU	Lichenized
OTU738	BSC	0,001	96.2%|FN868596|SH210044.07FU	Lichenized
OTU518	BSC	0,003	96.5%|KM245922|SH212345.07FU	Lichenized
OTU591	BSC	0,0288	97.3%|KC592271|SH212261.07FU	Lichenized
OTU1072	BSC	0,005	97.6%|HM624020|SH210034.07FU	Lichenized
OTU2	BSC	0,0004	97.8%|KR017059|SH204744.07FU	Lichenized
OTU468	BSC	0,0366	97.8%|KR017117|SH522867.07FU	Lichenized
OTU1521	BSC	0,049	98%|AY170792|SH210013.07FU	Lichenized
OTU223	BSC	0,0014	98.4%|AF455251|SH210057.07FU	Lichenized
OTU432	BSC	0,0004	98.4%|FJ536348|SH210027.07FU	Lichenized
OTU288	BSC	0,0008	98.6%|KF274213|SH200320.07FU	Lichenized
OTU2621	BSC	0,0006	99.3%|UDB024202|SH210064.07FU	Lichenized

OTU1107	BSC	0,01	99.6%|AF455243|SH032306.07FU	Lichenized
OTU467	BSC	0,0474	94.2%|JQ666431|SH016137.07FU	Litter saprotrophWood saprotroph
OTU1806	BSC	0,031	100%|JX322104|SH490836.07FU	Plant Pathogen
OTU303	BSC	0,0178	97.9%|KM186815|SH521472.07FU	Plant Pathogen
OTU1133	BSC	0,0002	98.8%|KF297266|SH462915.07FU	Plant pathogen
OTU523	BSC	0,0266	99.1%|KU687397|SH630073.07FU	Plant pathogen
OTU2633	BSC	0,036	90.2%|KC965434|SH004626.07FU	Plant saprotrophWood saprotroph
OTU97	BSC	0,0002	98.8%|HQ611327|SH004610.07FU	Plant saprotrophWood saprotroph
OTU29	BSC	0,002	99.6%|HQ211567|SH027387.07FU	PlantsaprotrophWood saprotroph
OTU28	BSC	0,0024	100%|KC965524|SH201645.07FU	Root associated fungus
OTU2838	BSC	0,0002	97.1%|KP889660|SH496534.07FU	Root associated fungus
OTU777	BSC	0,0026	99.6%|KC965515|SH205759.07FU	Root associated fungus
OTU247	BSC	0,0002	99.6%|KM504438|SH203237.07FU	Root associated fungus
OTU116	BSC	0,0042	100%|FJ475686|SH179956.07FU	Undefined saprotroph
OTU58	BSC	0,0424	100%|GQ907105|SH180000.07FU	Undefined saprotroph
OTU868	BSC	0,0462	100%|KM231845|SH182802.07FU	Undefined saprotroph
OTU587	BSC	0,0006	90.5%|HQ211764|SH181101.07FU	Undefined saprotroph
OTU2463	BSC	0,0064	90.5%|HQ211764|SH181101.07FU	Undefined saprotroph
OTU1187	BSC	0,0328	90.6%|AY394892|SH196478.07FU	Undefined saprotroph
OTU2480	BSC	0,037	91.1%|JF300498|SH219602.07FU	Undefined saprotroph
OTU2928	BSC	0,0048	91.4%|HQ611282|SH205752.07FU	Undefined saprotroph
OTU2149	BSC	0,038	91.7%|GU174404|SH020703.07FU	Undefined saprotroph
OTU700	BSC	0,0002	91.8%|KF274342|SH023354.07FU	Undefined saprotroph
OTU129	BSC	0,003	92.1%|HQ211650|SH181108.07FU	Undefined saprotroph
OTU148	BSC	0,0108	92.5%|HQ211764|SH181101.07FU	Undefined saprotroph
OTU1182	BSC	0,049	92.5%|JF449506|SH194265.07FU	Undefined saprotroph
OTU215	BSC	0,0002	92.7%|KC965292|SH006780.07FU	Undefined saprotroph
OTU647	BSC	0,0328	93.2%|KC966055|SH193298.07FU	Undefined saprotroph
OTU709	BSC	0,0336	93.3%|EF016381|SH193236.07FU	Undefined saprotroph
OTU2076	BSC	0,0314	93.5%|HQ611282|SH205752.07FU	Undefined saprotroph
OTU2157	BSC	0,0348	93.6%|EF016381|SH193236.07FU	Undefined saprotroph

OTU1017	BSC	0,033	94%|EF016381|SH193236.07FU	Undefined saprotroph
OTU458	BSC	0,0486	94.3%|JX857225|SH196236.07FU	Undefined saprotroph
OTU733	BSC	0,033	94.8%|EF016381|SH193236.07FU	Undefined saprotroph
OTU1099	BSC	0,0174	95.2%|KC966055|SH193298.07FU	Undefined saprotroph
OTU276	BSC	0,0036	95.3%|EF016381|SH193236.07FU	Undefined saprotroph
OTU76	BSC	0,005	96.2%|KP889343|SH493692.07FU	Undefined saprotroph
OTU3283	BSC	0,0146	96.3%|AY394892|SH196478.07FU	Undefined saprotroph
OTU109	BSC	0,0004	96.3%|FJ553668|SH201667.07FU	Undefined saprotroph
OTU140	BSC	0,0002	96.3%|KF274460|SH014744.07FU	Undefined saprotroph
OTU16	BSC	0,0072	96.3%|KT965054|SH522830.07FU	Undefined saprotroph
OTU96	BSC	0,0002	96.4%|EF016381|SH193236.07FU	Undefined saprotroph
OTU529	BSC	0,0128	96.6%|KF274317|SH181117.07FU	Undefined saprotroph
OTU1033	BSC	0,0356	96.6%|KP889978|SH493666.07FU	Undefined saprotroph
OTU582	BSC	0,0186	96.8%|GQ907105|SH180000.07FU	Undefined saprotroph
OTU250	BSC	0,0404	96.9%|EU292536|SH203866.07FU	Undefined saprotroph
OTU1068	BSC	0,0012	97.0%|FJ553294|SH193233.07FU	Undefined saprotroph
OTU6	BSC	0,0008	97.1%|KT965054|SH522830.07FU	Undefined saprotroph
OTU7	BSC	0,0012	97.4%|HQ260288|SH219619.07FU	Undefined saprotroph
OTU2811	BSC	0,03	97.5%|AB854726|SH196476.07FU	Undefined saprotroph
OTU641	BSC	0,0002	97.5%|FJ008679|SH193253.07FU	Undefined saprotroph
OTU327	BSC	0,0008	97.8%|HQ335299|SH223448.07FU	Undefined saprotroph
OTU1248	BSC	0,0002	98.1%|EF373581|SH193240.07FU	Undefined saprotroph
OTU2332	BSC	0,0004	98.8%|LC085188|SH193233.07FU	Undefined saprotroph
OTU386	BSC	0,0258	99.1%|KC965430|SH031254.07FU	Undefined saprotroph
OTU1762	BSC	0,0028	99.2%|HQ211764|SH181101.07FU	Undefined saprotroph
OTU629	BSC	0,0096	91.7%|HG937127|SH487800.07FU	
OTU727	BSC	0,002	91.9%|KF274293|SH017403.07FU	
OTU2360	BSC	0,033	92.1%|KF617587|SH215245.07FU	
OTU672	BSC	0,0004	92.4%|KF617295|SH456951.07FU	
OTU456	BSC	0,0004	93.3%|KP889786|SH208682.07FU	
OTU772	BSC	0,039	93.8%|KF274251|SH474472.07FU	
OTU125	BSC	0,0002	94.1%|KP889786|SH208682.07FU	

OTU117	BSC	0,004	94.7%|KF274293|SH017403.07FU	
OTU1915	BSC	0,0214	95.2%|KP889676|SH495465.07FU	
OTU256	BSC	0,0122	95.3%|JF300533|SH205580.07FU	
OTU663	BSC	0,0262	95.3%|KP889643|SH488454.07FU	
OTU236	BSC	0,0054	95.7%|KF274251|SH474472.07FU	
OTU595	BSC	0,0034	96.5%|KF617747|SH204499.07FU	
OTU60	BSC	0,0018	97.5%|DQ309156|SH202429.07FU	
OTU265	BSC	0,04	97.6%|GU174283|SH216436.07FU	
OTU486	BSC	0,0118	97.7%|JX998699|SH019200.07FU	
OTU567	BSC	0,0496	98.3%|KF617797|SH203042.07FU	
OTU64	BSC	0,014	98.8%|KF617982|SH027017.07FU	
OTU402	BSC	0,0192	99.5%|EF373561|SH200318.07FU	
OTU872	VV	0,0208	70.6%|KF028767|SH467878.07FU	
OTU2943	VV	0,049	70.9%|JQ666438|SH471875.07FU	
OTU390	VV	0,0476	71%|KF733464|SH494353.07FU	
OTU384	VV	0,0012	71.1%|JQ003630|SH223176.07FU	
OTU1221	VV	0,0014	71.1%|JQ003630|SH223176.07FU	
OTU798	VV	0,0054	71.1%|JQ003630|SH223176.07FU	
OTU1814	VV	0,0068	71.1%|JQ003630|SH223176.07FU	
OTU977	VV	0,009	71.1%|JQ003630|SH223176.07FU	
OTU1129	VV	0,0196	71.1%|JQ003630|SH223176.07FU	
OTU1088	VV	0,0294	71.1%|JQ003630|SH223176.07FU	
OTU482	VV	0,0424	71.1%|JQ003630|SH223176.07FU	
OTU2060	VV	0,0436	71.1%|JQ003630|SH223176.07FU	
OTU621	VV	0,0448	71.1%|JQ003630|SH223176.07FU	
OTU850	VV	0,0458	71.1%|JQ003630|SH223176.07FU	
OTU132	VV	0,0472	71.1%|JQ003630|SH223176.07FU	
OTU1162	VV	0,0034	72.3%|KF296953|SH485511.07FU	
OTU966	VV	0,0006	73.6%|JF300543|SH462390.07FU	
OTU442	VV	0,011	73.7%|AY219390|SH007649.07FU	
OTU1598	VV	0,0118	73.8%|KF297121|SH032388.07FU	

OTU1301	VV	0,0368	74.1%|EU002899|SH194513.07FU	
OTU960	VV	0,009	74.9%|JF300543|SH462390.07FU	
OTU438	VV	0,0124	75.5%|HG937143|SH497382.07FU	
OTU1371	VV	0,018	76%|JX456924|SH028220.07FU	
OTU1897	VV	0,034	76%|KF297121|SH032388.07FU	
OTU1635	VV	0,0154	76%|KF618164|SH482592.07FU	
OTU833	VV	0,0318	76.4%|KF800172|SH021105.07FU	
OTU123	VV	0,022	77.3%|KX228256|SH629393.07FU	
OTU1299	VV	0,0476	78.4%|UDB014634|SH217612.07FU	
OTU831	VV	0,0196	79.6%|FJ528734|SH001663.07FU	
OTU357	VV	0,001	80.2%|JF300543|SH462390.07FU	
OTU1004	VV	0,0464	80.3%|JX987750|SH474136.07FU	
OTU131	VV	0,0252	82.7%|HQ212111|SH190009.07FU	
OTU305	VV	0,0014	84.3%|KU534817|SH631256.07FU	
OTU1018	VV	0,042	86.8%|FN565212|SH217841.07FU	
OTU1889	VV	0,0064	87%|KC222820|SH180900.07FU	
OTU704	VV	0,0454	87.3%|LT799727|SH631030.07FU	
OTU183	VV	0,0482	87.9%|HM030614|SH000651.07FU	
OTU196	VV	0,002	89.2%|KC222820|SH180900.07FU	
OTU1142	VV	0,0496	89.3%|KP192607|SH524098.07FU	
OTU211	VV	0,0284	89.7%|KF617637|SH208638.07FU	
OTU2727	VV	0,044	89.8%|JQ761327|SH020836.07FU	
OTU514	VV	0,0218	90%|EF434092|SH467192.07FU	
OTU283	VV	0,0012	94%|KP889666|SH493700.07FU	
OTU112	VV	0,02	95%|JQ420967|SH179997.07FU	
OTU485	VV	0,0016	95%|JQ666464|SH001662.07FU	
OTU879	VV	0,0346	96%|KF296822|SH016383.07FU	
OTU2425	VV	0,0084	97%|HQ829336|SH204501.07FU	
OTU42	VV	0,0004	100%|JN049844|SH184933.07FU	Animal Pathogen
OTU899	VV	0,0432	97.8%|KF309965|SH206048.07FU	Animal Pathogen
OTU788	VV	0,036	98.8%|JX981910|SH183188.07FU	Animal Pathogen
OTU1025	VV	0,0268	98.7%|HQ211494|SH201714.07FU	Ectomycorrhiza

OTU70	VV	0,0014	100%|KC702643|SH214657.07FU	Ectomycorrhizal
OTU1902	VV	0,0422	100%|LN850503|SH181005.07FU	Ectomycorrhizal
OTU154	VV	0,026	90.8%|UDB002308|SH194441.07FU	Ectomycorrhizal
OTU3151	VV	0,0468	92.9%|AY078145|SH275913.07FU	Ectomycorrhizal
OTU2436	VV	0,0492	95.6%|AY078145|SH275913.07FU	Ectomycorrhizal
OTU819	VV	0,0056	98.2%|UDB017965|SH227199.07FU	Ectomycorrhizal
OTU229	VV	0,0382	98.4%|JN032545|SH028720.07FU	Ectomycorrhizal
OTU861	VV	0,0064	98.9%|UDB000350|SH176549.07FU	Ectomycorrhizal
OTU1219	VV	0,0386	99.2%|KC965984|SH214290.07FU	Ectomycorrhizal
OTU2014	VV	0,0296	96.4%|DQ309187|SH221487.07FU	Ericoid mycorrhizal
OTU1023	VV	0,0046	99.2%|FJ554384|SH174320.07FU	Ericoid mycorrhizal
OTU501	VV	0,0012	99.2%|KF617682|SH012952.07FU	Ericoid mycorrhizal
OTU919	VV	0,0176	99.5%|KC019909|SH183303.07FU	Ericoid mycorrhizal
OTU590	VV	0,0202	94.9%|AF444362|SH206653.07FU	Fungal parasite
OTU539	VV	0,0032	99.6%|HQ211717|SH193767.07FU	Fungal parasite
OTU381	VV	0,0002	100%|HM589383|SH185977.07FU	Litter saprotrophUndefined saprotroph
OTU30	VV	0,0002	100%|HQ211581|SH187862.07FU	Litter saprotrophUndefined saprotroph
OTU77	VV	0,014	100%|HQ212268|SH180114.07FU	Litter saprotrophUndefined saprotroph
OTU296	VV	0,0144	100%|HQ445990|SH180130.07FU	Litter saprotrophUndefined saprotroph
OTU944	VV	0,0064	92.1%|KP889624|SH198290.07FU	Litter saprotrophUndefined saprotroph
OTU1548	VV	0,0364	95.1%|EF152531|SH187874.07FU	Litter saprotrophUndefined saprotroph
OTU526	VV	0,0008	95.4%|HQ211500|SH185974.07FU	Litter saprotrophUndefined saprotroph
OTU337	VV	0,0254	96.4%|EF152531|SH187874.07FU	Litter saprotrophUndefined saprotroph
OTU628	VV	0,0002	97%|HQ211581|SH187862.07FU	Litter saprotrophUndefined saprotroph
OTU2141	VV	0,0412	97%|HQ211581|SH187862.07FU	Litter saprotrophUndefined saprotroph
OTU1255	VV	0,0402	99.1%|HQ212285|SH003974.07FU	Litter saprotrophUndefined saprotroph
OTU1	VV	0,034	99.1%|KJ735026|SH495966.07FU	Litter saprotrophUndefined saprotroph
OTU32	VV	0,0002	99.6%|HQ211515|SH185973.07FU	Litter saprotrophUndefined saprotroph
OTU620	VV	0,01	99.6%|JF300755|SH185976.07FU	Litter saprotrophUndefined saprotroph
OTU661	VV	0,0006	91.9%|AY626983|SH200714.07FU	Plant pathogen
OTU155	VV	0,0042	99.5%|KM186810|SH524027.07FU	Plant Pathogen

OTU65	VV	0,0008	100%|FJ475732|SH196224.07FU	Plant saprotrophWood saprotroph
OTU261	VV	0,0076	100%|HQ211754|SH004617.07FU	Plant saprotrophWood saprotroph
OTU379	VV	0,0288	100%|KC876277|SH214271.07FU	Plant saprotrophWood saprotroph
OTU135	VV	0,001	99.6%|KF617352|SH214273.07FU	Plant saprotrophWood saprotroph
OTU268	VV	0,0026	94.3%|KP866123|SH201717.07FU	Root associated fungus
OTU55	VV	0,003	95.1%|KP889660|SH496534.07FU	Root associated fungus
OTU3102	VV	0,0352	95.5%|KP889660|SH496534.07FU	Root associated fungus
OTU991	VV	0,0066	100%|AB476491|SH005167.07FU	Undefined saprotroph
OTU37	VV	0,0102	100%|AF444350|SH190017.07FU	Undefined saprotroph
OTU204	VV	0,0004	100%|FJ553238|SH193764.07FU	Undefined saprotroph
OTU396	VV	0,0106	100%|HM069375|SH024117.07FU	Undefined saprotroph
OTU284	VV	0,0046	100%|KC965124|SH219520.07FU	Undefined saprotroph
OTU2013	VV	0,008	100%|KF296912|SH205446.07FU	Undefined saprotroph
OTU171	VV	0,0002	100%|KT923624|SH201431.07FU	Undefined saprotroph
OTU1656	VV	0,0474	91.6%|HQ211529|SH005170.07FU	Undefined saprotroph
OTU220	VV	0,0188	92.1%|FN663743|SH105367.07FU	Undefined saprotroph
OTU843	VV	0,0056	94.1%|KF618164|SH482592.07FU	Undefined saprotroph
OTU2450	VV	0,0014	94.5%|JN032572|SH203828.07FU	Undefined saprotroph
OTU2757	VV	0,04	94.7%|GU062199|SH018514.07FU	Undefined saprotroph
OTU82	VV	0,0152	95.3%|KC965670|SH193252.07FU	Undefined saprotroph
OTU218	VV	0,007	95.4%|DQ309141|SH179085.07FU	Undefined saprotroph
OTU156	VV	0,004	95.6%|KF823592|SH208055.07FU	Undefined saprotroph
OTU1227	VV	0,011	96.6%|HM036653|SH180664.07FU	Undefined saprotroph
OTU1482	VV	0,0206	96.6%|HM230874|SH181130.07FU	Undefined saprotroph
OTU858	VV	0,0014	96.8%|KM504451|SH494719.07FU	Undefined saprotroph
OTU93	VV	0,027	97.5%|AB854709|SH496664.07FU	Undefined saprotroph
OTU2349	VV	0,02	97.5%|JX981884|SH110708.07FU	Undefined saprotroph
OTU447	VV	0,024	97.6%|KF285997|SH207163.07FU	Undefined saprotroph
OTU401	VV	0,0016	97.8%|AB854709|SH496664.07FU	Undefined saprotroph
OTU1385	VV	0,0394	97.8%|HM230874|SH181130.07FU	Undefined saprotroph
OTU506	VV	0,0002	98.4%|HQ611282|SH205752.07FU	Undefined saprotroph
OTU4	VV	0,0002	98.5%|EU292507|SH203829.07FU	Undefined saprotroph

OTU857	VV	0,0416	98.6%|KC411730|SH207159.07FU	Undefined saprotroph
OTU1677	VV	0,0444	98.8%|HM069367|SH192597.07FU	Undefined saprotroph
OTU54	VV	0,0028	98.8%|KM504451|SH494719.07FU	Undefined saprotroph
OTU186	VV	0,0012	98.9%|KF617378|SH005082.07FU	Undefined saprotroph
OTU143	VV	0,0002	99.6%|HQ211812|SH213411.07FU	Undefined saprotroph
OTU1273	VV	0,0262	99.7%|KP889727|SH488438.07FU	Undefined saprotroph
OTU92	VV	0,0318	99.5%|JN021035|SH208316.07FU	Undefined saprotrophUndefined Biotroph
OTU1764	VV	0,0454	100%|KP814424|SH202375.07FU	Wood saprotroph
OTU699	VV	0,0242	100%|EF521253|SH004454.07FU	
OTU201	VV	0,0018	100%|KF617492|SH006003.07FU	
OTU176	VV	0,028	90.7%|KF617628|SH023327.07FU	
OTU551	VV	0,0142	91.2%|HG937127|SH487800.07FU	
OTU1730	VV	0,0174	92.8%|KF617532|SH473856.07FU	
OTU1499	VV	0,0122	93.2%|KF617532|SH473856.07FU	
OTU614	VV	0,0004	95.6%|JX981914|SH463853.07FU	
OTU251	VV	0,001	95.7%|EU292495|SH208584.07FU	
OTU2760	VV	0,0476	95.9%|JQ666464|SH001662.07FU	
OTU862	VV	0,0074	95.9%|JX363263|SH526435.07FU	
OTU445	VV	0,0002	96.4%|JQ666464|SH001662.07FU	
OTU711	VV	0,004	96.4%|KF660575|SH487547.07FU	
OTU1715	VV	0,0002	96.5%|FJ475780|SH204497.07FU	
OTU56	VV	0,0002	96.9%|EU292495|SH208584.07FU	
OTU440	VV	0,007	97.2%|EU292495|SH208584.07FU	
OTU1144	VV	0,049	97.6%|UDB014664|SH531926.07FU	
OTU1207	VV	0,0234	98.1%|KF617295|SH456951.07FU	
OTU611	VV	0,0092	98.4%|KC222820|SH180900.07FU	
OTU478	VV	0,0056	98.5%|EU292330|SH204504.07FU	
OTU1595	VV	0,0102	98.5%|KP889572|SH492688.07FU	
OTU555	VV	0,0004	98.6%|JQ666464|SH001662.07FU	
OTU766	VV	0,0018	99.1%|KX609411|SH218810.07FU	
OTU742	VV	0,0024	99.5%|JN032559|SH219501.07FU	

OTU1740	VV	0,0194	99.7%|KP889455|SH495473.07FU	

Table S3 Regression slopes and r^2 for the variation of relative richness and abundance of the total fungal community and eight functional guilds in response to soil physicochemical properties (significant for p<0,05; [§]marginally significant, p<0.1; n. s. non-significant).

		Water content (%)		pH		P content (mg/kg)		C content (%)		N content (%)		C/N ratio	
		slope	r^2	slope	r^2	slope	r^2	slope	r^2	slope	r^2	slope	r^2
All fungi	Richness	1.1083	0.1259	-40.19	0.0745	0.1834	0.0557	n. s.		n. s.		n. s.	
ECM fungi	Richness	0.0918	0.0892	n. s.		n. s.		n. s.		n. s.		n. s.	
	Abundance	n. s.		8.272	0.0600	-0.035[§]	0.0362	-0.2665	0.0611	-5.949[§]	0.0350	-0.5419	0.0619
ERM fungi	Richness	0.0389[§]	0.0347	-3.1287	0.1471	0.0094[§]	0.0394	0.0629[§]	0.0474	1.5113[§]	0.0328	0.1254[§]	0.0457
	Abundance	0.0232[§]	0.0350	-1.357	0.0702	0.0068	0.0675	0.0491	0.0949	1.5762	0.1376	n. s.	
Other root associated fungi	Richness	n. s.		-1.7431	0.0635	0.0069[§]	0.0313	n. s.		n. s.		n. s.	
	Abundance	n. s.		-1.7180	0.108	n. s.		0.0477	0.0771	n. s.		0.1105	0.1065
Lichenized fungi	Richness	-0.1034	0.1387	n. s.		n. s.		n. s.		n. s.		0.1776[§]	0.0362
	Abundance	n. s.		n. s.		n. s.		n. s.		n. s.		n. s.	
Plant pathogens	Richness	0.0654	0.1026	-2.602	0.0752	n. s.		n. s.		n. s.		n. s.	
	Abundance	-0.0497[§]	0.0425	n. s.		n. s.		n. s.		n. s.		n. s.	
Animal pathogens	Richness	n. s.		-4.1856	0.3929	0.0172	0.2468	0.1157	0.2888	2.7649	0.2171	0.2231	0.2611
	Abundance	n. s.		n. s.		n. s.		0.0760	0.0626	1.9074	0.0502	n. s.	
Mycoparasites	Richness	0.0234[§]	0.0309	-1.8274	0.1261	0.0064	0.0508	n. s.		0.9206[§]	0.0302	0.0905	0.0665
	Abundance	n. s.		-0.3437	0.1252	n. s.		n. s.		n. s.		0.0176	0.0719
Saprotrophs	Richness	0.5974	0.2467	-19.081	0.1141	0.0890	0.0920	n. s.		10.970	0.0394	0.8194[§]	0.0404
	Abundance	0.1862[§]	0.0481	n. s.		n. s.		n. s.		n. s.		n. s.	

Table S4 Regression r^2 and significance of each variable fitted in the NMDS analyses (fig. 5).

Variables	**All fungi**		**ECM fungi**		**ERM fungi**		**Other root associated fungi**		**Lichenized fungi**	
	r^2	*p*	r^2	*p*	r^2	*p*	r^2	*p*	r^2	*p*
pH	0.6865	0.001	0.7359	0.001	0.3998	0.001	0.3626	0.001	0.2952	0.001
Water content	0.4767	0.001	0.1867	0.005	0.2726	0.002	0.1716	0.008	0.0796	0.109
C	0.5326	0.001	0.5603	0.001	0.2493	0.001	0.2634	0.001	0.1406	0.019
N	0.4577	0.001	0.4943	0.001	0.1811	0.006	0.2071	0.001	0.1212	0.034
C/N ratio	0.6042	0.001	0.6029	0.001	0.3840	0.001	0.3134	0.001	0.1761	0.013
P	0.3728	0.001	0.5017	0.001	0.1629	0.007	0.1807	0.004	0.1400	0.019
Salix	0.2190	0.002	0.1446	0.008	0.1841	0.007	0.0871	0.078	0.1674	0.008
Betula	0.0508	0.248	0.3246	0.001	0.0355	0.351	0.0100	0.748	0.0207	0.595
Vaccinium	0.2784	0.001	0.0529	0.211	0.0817	0.083	0.0356	0.378	0.0483	0.261
Empetrum	0.0679	0.136	0.0597	0.162	0.1141	0.031	0.0833	0.100	0.1428	0.020

Variables	**Plant pathogens**		**Animal pathogens**		**Mycoparasites**		**Saprotrophs**	
	r^2	*p*	r^2	*p*	r^2	*p*	r^2	*p*
pH	0.5607	0.001	0.4356	0.001	0.1049	0.046	0.6276	0.001
Water content	0.2351	0.002	0.4195	0.001	0.1059	0.051	0.3656	0.001
C	0.4804	0.001	0.4460	0.001	0.0755	0.106	0.5499	0.001
N	0.4713	0.001	0.3734	0.001	0.0708	0.119	0.4351	0.001
C/N ratio	0.4630	0.001	0.4641	0.001	0.1290	0.021	0.6324	0.001
P	0.3553	0.001	0.3439	0.001	0.0100	0.745	0.3322	0.001
Salix	0.2009	0.006	0.1719	0.001	0.1100	0.060	0.1767	0.001
Betula	0.0506	0.233	0.0107	0.716	0.0148	0.660	0.0325	0.355
Vaccinium	0.2631	0.001	0.2909	0.001	0.0884	0.083	0.1653	0.004
Empetrum	0.0113	0.738	0.0818	0.095	0.0016	0.947	0.0842	0.081
